# Bi‐Functional Diaminopropane Additive Enables Stable Li Anodes and Highly Efficient Cathodes for High‐Performance Li‐Air Batteries

**DOI:** 10.1002/advs.202505539

**Published:** 2025-05-29

**Authors:** Honghao Hu, Qingxu Zhang, Jiucong Liu, Junjie Li, Pingli Wu, Caicai Li, Peipei Du, Huiqiao Li, Xizheng Liu

**Affiliations:** ^1^ Key Laboratory of Flexible Optoelectronic Materials and Technology Ministry of Education School of Optoelectronic Materials & Technology Jianghan University Wuhan 430056 China; ^2^ Institute for New Energy Materials and Low‐Carbon Technologies School of Materials Science and Engineering Tianjin University of Technology Tianjin 300384 China; ^3^ State Key Laboratory of Materials Processing and Die & Mould Technology School of Materials Science and Engineering Huazhong University of Science and Technology Wuhan 430074 China

**Keywords:** bi‐functional additive, diaminopropane, in situ gel layer, Li‐air batteries, redox mediators

## Abstract

Low round‐trip efficiency and limited cycle durability remain significant challenges for commercial of Lithium‐air batteries (LABs). This study introduces a novel electrolyte additive, diaminopropane (DAP), that simultaneously addresses anode stability and cathode reaction kinetics in ambient LABs. The proposed mechanism involves a bi‐function approach: 1) at the anode, DAP spontaneously reacts with metallic Li to form Li‐DAP, which subsequently cross‐links with ether solvents to create a protective gel layer. This layer effectively mitigates Li dendrite formation and shields the anode from ambient moisture and CO_2_ corrosion. 2) At the cathode, DAP modifies the oxygen reduction pathway from surface‐mediated to solution‐mediated, while simultaneously acting as an efficient redox mediator during charging. This dual functionality results in a remarkable reduction of the initial charging potential from 4.2 to 3.4 V, accompanied by observed singlet oxygen quenching. The implemented DAP additive enables LABs to achieve unprecedented cycling stability, demonstrating continuous operation for 1000 h in ambient air while maintaining energy efficiency exceeding 70%. This work establishes an effective electrolyte additive strategy for developing high‐performance ambient LABs through simultaneous anode protection and enhanced cathode reaction reversibility.

## Graphical abstract

Diaminopropane (DAP) solvent is employed as a bi‐functional additive for Li‐air batteries. It spontaneously reacts to form a gel layer to prevent corrosion on the Li anode and maintain a liquid state at the cathode to improve the decomposition of Li_2_O_2_. This dual mechanism provides critical insights for the development of practical, ambient‐operable LABs.

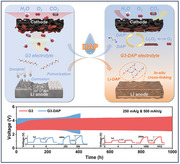

## 1 Introduction

The rapid development of electric vehicles and aircraft has created growing demand for batteries with higher energy densities. As traditional Li‐ion batteries are approaching their theoretical capacity limits, there is an urgent need to develop new secondary batteries with superior energy density to meet these demanding requirements.^[^ ^1^ ^]^ The LABs have emerged as one of the most promising candidates due to their ultra‐high theoretical energy density (3500 Wh kg^−1^), which is achieved by utilizing oxygen from the ambient environment as the active component at the cathode.^[^ ^2^ ^]^ However, two major challenges hinder their practical application: first, the corrosion of the Li anode in ambient air significantly deteriorates cycling performance; second, sluggish electrode reaction kinetics at the cathode lead to substantial polarization and low round‐trip efficiency.^[^ ^3^ ^]^

Feasible strategies to mitigate metallic Li anode corrosion from moisture and CO_2_ exposure in open battery systems primarily focus on electrolyte structure optimization. Solid‐state electrolytes could effectively prevent moisture and CO_2_ penetration to the Li anode, thereby inhibiting corrosion and enhancing stability.^[^ ^4^ ^]^ However, their practical implementation is constrained by low ionic conductivity. Gel polymer electrolytes, which integrate the superior mechanical properties of polymer matrices with the high ionic conductivity of liquid electrolytes, present a viable solution to these limitations.^[^ ^5^ ^]^ Despite these advantages, challenges persist, including inadequate electrode‐electrolyte interfacial contact and significant ion diffusion resistance. In situ polymerized gel electrolytes, maintaining the interfacial characteristics of liquid electrolytes while adhering to solid electrodes, demonstrate excellent compatibility with Li anode surfaces.^[^ ^6^ ^]^ Cao et al.^[^ ^7^ ^]^ developed a gel polymer electrolyte through in situ polymerization of 1,3‐dioxolane (DOL), effectively protecting Li anodes in LABs. This approach, enhanced by the protective gradient SEI film, enabled LABs to achieve an extended cycle life of 170 cycles. Our research group has advanced this technology by introducing an in situ cross‐linked gel electrolyte utilizing Li‐EDA and ether‐based solvents, which facilitated a remarkable cycling life of 1200 h for LABs operating in ambient air conditions.^[^ ^8^ ^]^ These advancements have significantly extended LABs' cycling durability and enabled their operation in atmospheric environments. Nevertheless, complete electrolyte gelation presents new challenges, particularly the obstruction of gas diffusion channels at the cathode, potentially compromising cathode reaction kinetics. Additionally, the low conductivity of discharge products generates substantial charge transfer resistance at the gel electrolyte‐cathode interface, dynamically restricting Li^+^ transport across the three‐phase boundary.^[^ ^9^ ^]^ These emerging issues contribute to increased overpotentials, reduced cycling stability, and diminished energy efficiency. Consequently, the development of efficient electrolytes and optimization of cathode/gel electrolyte interfaces have become paramount for realizing practical LABs applications.

The inherently low ionic conductivity of discharge products at the cathode typically leads to the substantial charge overpotential and reduced energy efficiency. Furthermore, the inadequate interfacial contact between discharge products, gel electrolyte, and catalyst at the cathode exacerbates the charging efficiency, ultimately resulting in suboptimal cycling performance of LABs.^[^ ^10^ ^]^ Recent advancements have demonstrated the efficacy of various redox mediators (RMs), including LiI, tetrathiafulvalene (TTF), and 2,5‐di‐tert‐butyl‐1,4‐benzoquinone (DBBQ), and others.^[^ ^11^ ^]^ These RMs not only facilitate reduced charging overpotentials but also effectively suppress the generation of singlet oxygen (^1^O_2_).^[^ ^12^ ^]^ It has been established that highly reactive ^1^O_2_, generated during the discharge process, readily degrades various cell components, including organic solvents, electrolyte salts, and electrodes. This degradation leads to the formation of passivating by‐products, causing capacity fade and potentially premature battery failure.^[^ ^13^ ^]^ However, the implementation of RMs presents a significant challenge: their tendency to migrate to the anode, resulting in metallic Li corrosion, which has hindered their practical application in liquid‐electrolyte‐based LABs.^[^ ^14^ ^]^ While the integration of RMs with in situ gel electrolytes has shown promise in mitigating Li corrosion, this approach introduces another complication: the complete gelated electrolyte impedes oxygen diffusion.^[^ ^15^ ^]^ Consequently, the primary research focus has shifted toward developing strategies that prevent RM migration to the anode while maintaining a liquid state at the cathode, representing a critical challenge in LABs optimization.

**Figure 1:**
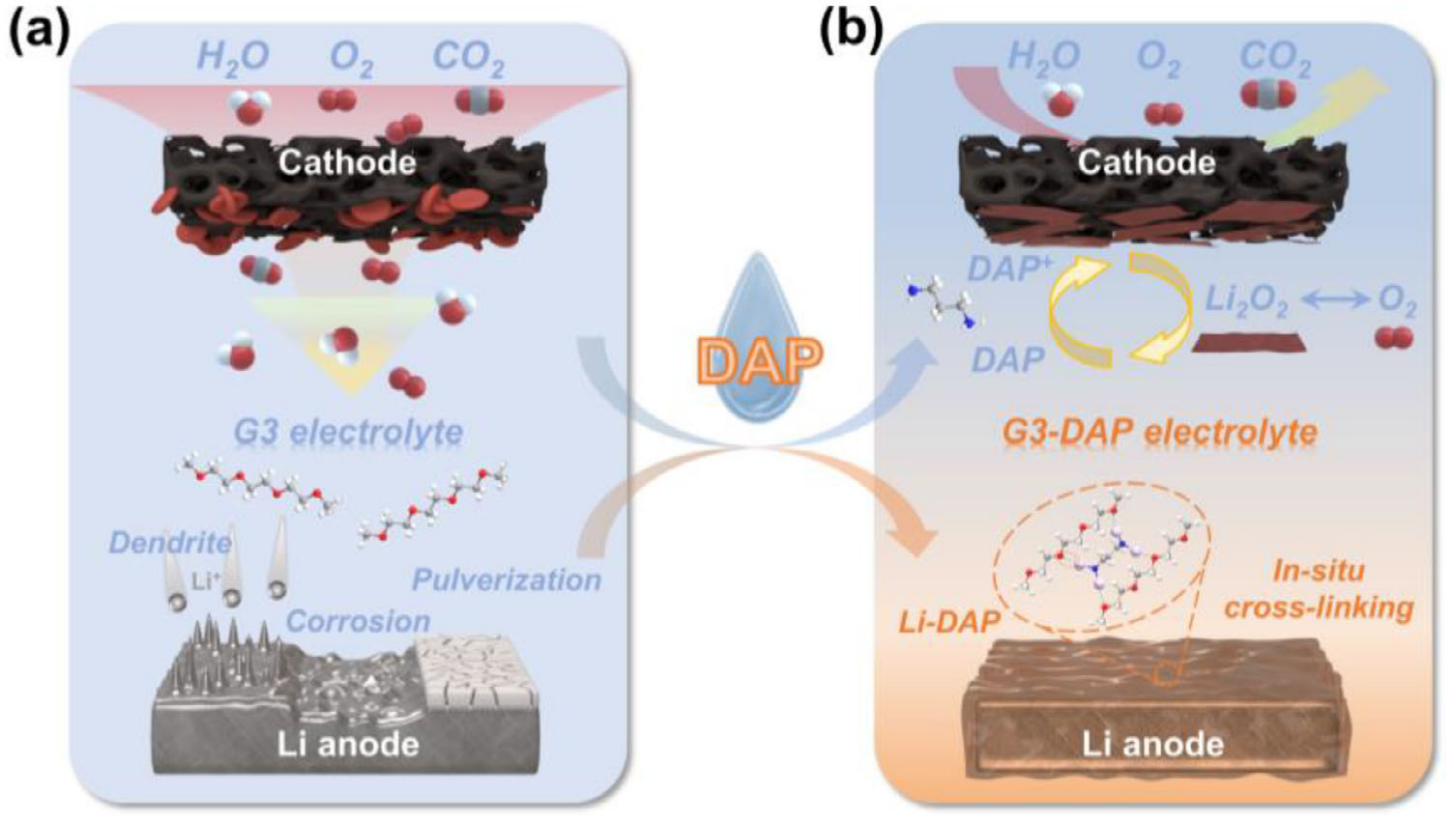
Schematic illustration the challenges for conventional LABs with (a) normal liquid electrolyte and (b) effects of DAP electrolyte additive in LABs, mediate reactions at cathode and in situ electrolyte gelation at anode.

In this study, we introduce a novel electrolyte additive, organic amine DAP, for ambient LABs. The spontaneous formation of Li‐DAP at the Li anode surface subsequently cross‐links with ether solvents to create a thin gel layer. Simultaneously, DAP maintains its liquid state at the cathode, functioning as an effective redox mediator to enhance the decomposition of discharge products during the charging process (**Figure** **1**). Theoretical calculations reveal that the redox potential of DAP exhibits excellent compatibility with O_2_ reduction/evolution potentials, thereby demonstrating its efficacy as a redox mediator at the cathode. Comprehensive gas‐diffusion experiments, including calendar lifespan assessments and gas chromatography analyses, confirm the gel electrolyte's ability to effectively suppress moisture and CO_2_ diffusion to the Li anode, significantly delaying anode corrosion. Furthermore, we have verified the quenching of ^1^O_2_ and the modification of the O_2_ reduction pathway during discharge. LABs incorporating the DAP additive achieve remarkable performance metrics, including a cycle life exceeding 1000 h in ambient air conditions. The implementation of DAP reduces charging overpotentials from 4.2 to 3.4 V, achieving an energy efficiency of 70%. This work provides fundamental insights into the multifunctional role of organic amines as electrolyte additives in LABs and establishes a new paradigm for developing long‐cycling, high‐efficiency LABs capable of operating directly in ambient conditions.

## 2 Results and Discussion

With normal liquid electrolyte in LABs (Figure 1a), possible dendritic growth and Li anode corrosion by moisture and CO_2_, electrolyte volatilization, and the formation of high crystallinity discharge products in the cathode always deteriorate their performance. By introducing DAP additive, as shown in Figure 1b, gradient gel electrolyte is in situly formed at the surface of Li anode during the rest of assembled batteries. The organic amine can react with metallic Li to form an amino lithium, and the amino lithium molecule cross linked with ether molecule to form a gel layer. This gel layer can protect the Li anode from corrosion by moisture and CO_2_ in the ambient air. In our previous work,^[^ ^8^ ^]^ ethanediamine can initiate the gelation of ether electrolyte in a short time, but high reactivity with metallic Li usually leads to completely gelation of electrolyte and successive Li corrosion. Replacing by DAP, the lower Li reaction ability and long chain structure lead to weak gelation ability with ether solvent, in which gradient gel formed at the Li surface. Near the cathode, organic amine DAP remains liquid state, and could participate in the oxygen reduction/evolution reaction, promoting the decomposition of discharged products during the OER process. The corresponding optical photos of the ex situ gelation process of organic amine and ether‐based solvent are shown in Figures S1 and S2 (Supporting Information). Interestingly, with lower proportion of DAP, it is difficult to gelatinization even after a long‐time rest, which indicates the moderate reaction activity of DAP and metallic Li. This means the Li anode would be stable even after long term cycling with DAP additive. Meanwhile, the violent reactions between Li and EDA result in quickly gelation and successive Li corrosion if there is excess amount of EDA. This is in consistent with the results of calculated Gibbs free energy changes (ΔG) of ethylenediamine (EDA), propylene diamine (DAP), diethylenetriamine (DETA) reacting with Li metal as shown in Figure S3 (Supporting Information). Linear sweep voltammetry (LSV) was used to study the electrochemical stability of different gel groups and compared with liquid electrolytes, as shown in Figure S4 (Supporting Information), the onset potential of gel electrolytes is higher than that of corresponding liquid counterparts by ≈ 0.5 V, indicating the enhanced electrochemical stability. The Li^+^ transference number of five different gel groups around G3 and DAP were calculated, and the G3‐DAP gel electrolyte demonstrates the highest Li^+^ transference number of different gel groups (Figure S5, Supporting Information). This means the suppressed transportation of TFSI^−^ to some extent.^[^ ^16^ ^]^ Fourier transform infrared spectroscopy (FTIR) analysis revealed that within the range of 500‐400 cm^−1^, a distinct and relatively prominent peak can be observed, which corresponds to the formation of Li‐O bonds, demonstrating the internal cross‐linking of the solvent and formation of the gel layer (Figure S6, Supporting Information). The stretching vibration peaks corresponding to NH‐ can also been observed in the range of 1700–1600 cm^−1^, which distinct the presence of organic amine components in the gel layer. The decrease in ionic conductivity of the G3‐DAP gel electrolyte is primarily due to the in situ crosslinking of the electrolyte. In contrast to lithium anode protection, the ionic conductivity does not significantly affect the cycling performance of the LABs (Figure S7, Supporting Information).

**Figure 2:**
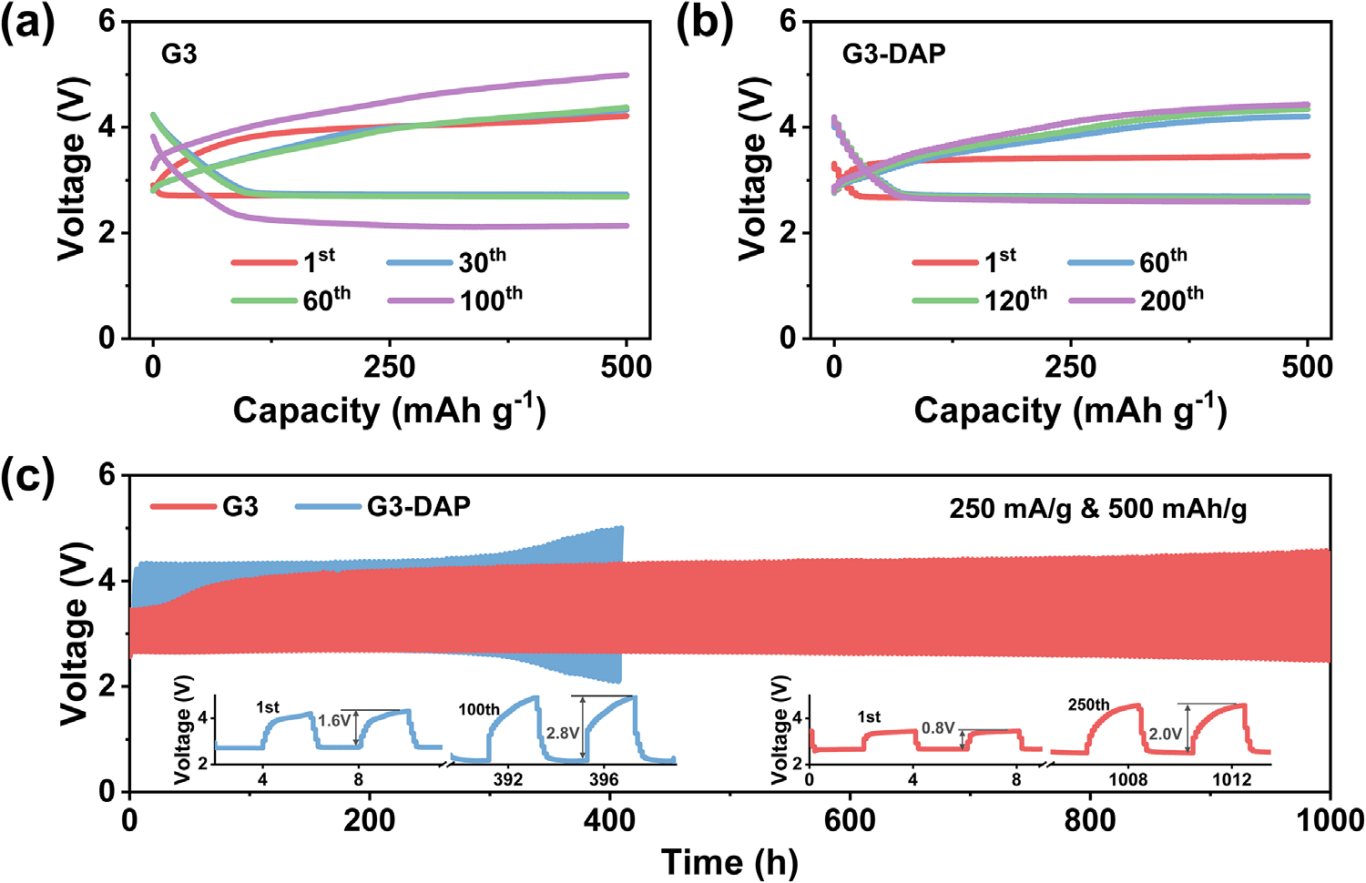
Electrochemical performance of LABs with different electrolytes. Discharge and charge profiles of the Li‐air batteries (a) without and (b) with DAP additives at 250 mA g^−1^. c) Cycling performance of ambient LABs with G3 and G3‐DAP electrolytes in ambient air.

Systematic screening of ether solvents with different molecular weight (G4, G3, G2) and organic amine additives (EDA, DAP, DETA) have been carried out. Figures S8 and S9 (Supporting Information) show the cycling performance of LABs with different electrolyte. Compared with pure liquid ether electrolyte, LABs with amine additives demonstrate a significantly extended the cycle life of LABs. Among them, the LABs with G3‐DAP gel electrolyte display the best cycling stability. In addition, G4 gel electrolyte LABs show similar cycle performance to G3 gel electrolyte, that is, the cycle life of G4‐DAP gel is better than other components, and it can be stably cycled for more than 500 h. However, its energy efficiency is slightly lower than that of G3‐DAP. Specifically, with G2 as solvent in electrolyte, due to its low molecular weight and highly volatility, no matter gel electrolyte is formed or not, the cell death caused by solvent volatilization only after ≈ 20 h (Figure S10, Supporting Information). Therefore, the G3 has been selected as ether solvent in the following experiments. The amount of DAP additive has also been optimized according to the LABs cycling performance, and 2 vol.% DAP additive displays a better performance (Figure S11, Supporting Information), and this electrolyte composition was chosen for the following experiment. **Figure** **2**a provide a more detailed demonstration of the long cycle performance with this electrolyte. In LABs, after circulating the G3 liquid electrolyte for ≈400 h, the charging potential exceeds 5 V, leading to sudden battery failure. In contrast, under the same conditions, the LABs with G3‐DAP gradient gel electrolyte demonstrate consistent discharge/charge curves for over 200 cycles (Figure 2b). The selected initial and last two cycles of voltage profiles are shown inner Figure 2c. The LABs with G3 electrolyte have a discharge platform that decays from initial 2.7 to 2.1 V before and after long cycles, and a charging platform that polarizes from 4.0 to 4.4 V. On the contrary, after the introduction of DAP additive, the discharge platform of LABs with G3‐DAP electrolyte before and after cycling only decayed from 2.7 to 2.5 V, and the charging platform also slowly increased from 3.1 to 4.1 V. As shown in Figure S12 (Supporting Information), the LABs with G3‐DAP electrolyte exhibited low charge overpotentials across various current densities. At 100 mA g^−1^, the charge overpotential was only 0.49 V. Even under high current of 1000 mA g^−1^, the overpotential remained stable at 1.27 V, significantly lower than that of the G3 electrolyte (1.43 V). Furthermore, the cycled Li anode was examined, revealing that the Li anode surface in batteries assembled with G3 electrolyte exhibited significant corrosion after cycling, appearing white with lithium hydroxide as the primary component. In contrast, batteries assembled with G3‐DAP electrolyte formed an in situ gel layer on the Li anode surface during cycling, resulting in a darkened electrode surface. These results indicate that DAP effectively enhances the oxygen reaction kinetics on the cathode side and mitigates Li anode corrosion, thereby significantly improving the cycling stability of LABs. The cycling performance is among one of the best performances as shown in Table S1 (Supporting Information). The Li//Li symmetric cells were also assembled to investigate the stability of electrolyte (Figure S13, Supporting Information). At the beginning, there is only slight difference in overvoltage between G3 liquid electrolyte and G3‐DAP gradient gel electrolyte, both are ≈ 30 mV. As Li^+^ deposition and stripping continues, with liquid electrolyte, there is a gradual increase of electrode polarization after only 130 h and a sudden surge in voltage occurs at the 170 h, which can be ascribed to short circuiting by Li dendrites. Under the protection of G3‐DAP gradient gel electrolyte, amine lithium layer formed in situly on the surface of the Li anode surface which can effectively inhibit the growth of Li dendrites, and thus cycling performance has been extended to more than 400 h.

**Figure 3:**
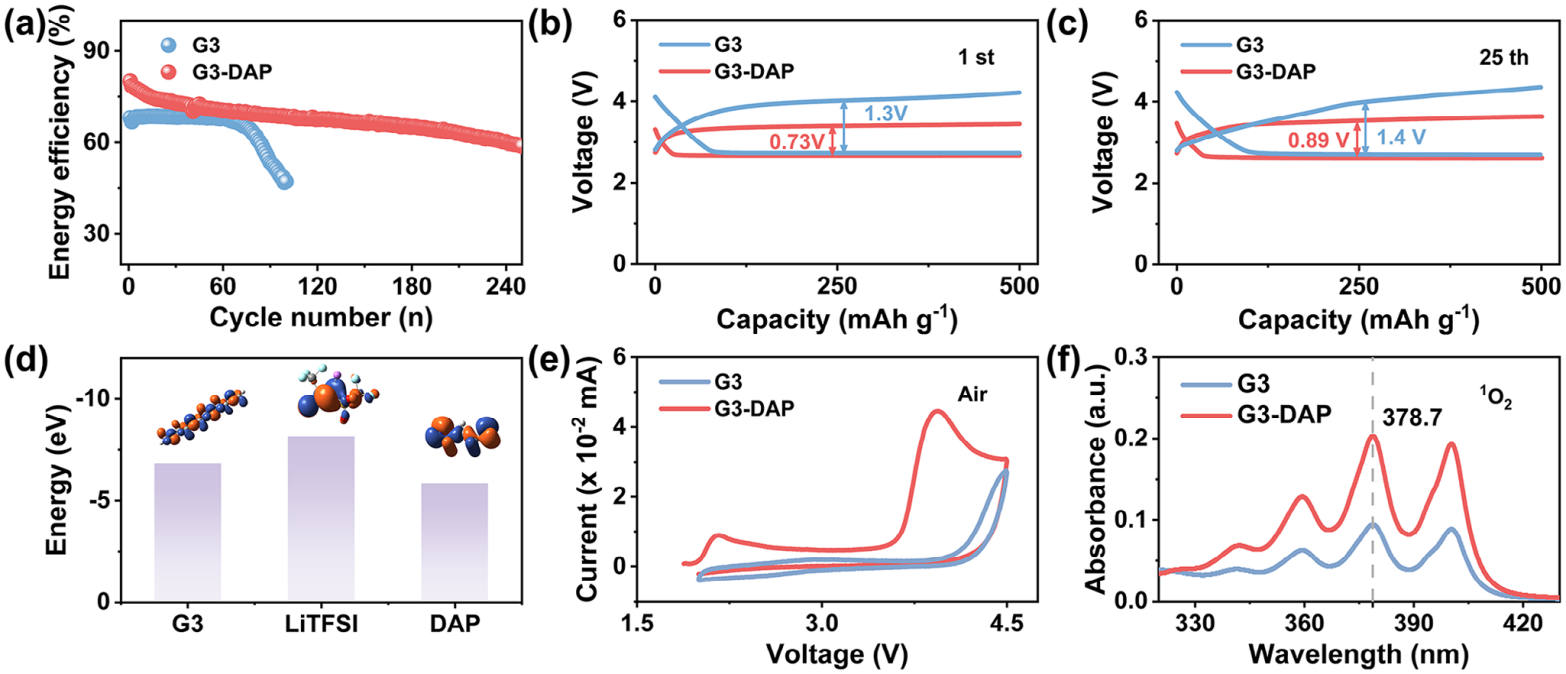
The working mechanism of DAP in LABs. a) Energy efficiency of ambient LABs with G3 liquid electrolyte and G3‐DAP electrolyte. Discharge and charge profiles of the LABs at the b) 1^st^ and the c) 25^th^ cycle. d) The calculated HOMO energy of G3, LiTFSI and DAP molecular. e) CV curves of batteries with and without DAP at a scanning rate of 0.1 mV s^−1^. f) Ultraviolet‐visible absorption spectrum of DMA in contact with a disassembled cathode discharged in G3 electrolyte and G3‐DAP electrolyte to detect the ^1^O_2_.

The formation and working mechanism of G3‐DAP gradient gel electrolyte have been systematically studied. The energy efficiency of the LABs with two different electrolytes was shown in **Figure** **3**a. Under a current density of 250 mA g^−1^ with a limited capacity of 500 mAh g^−1^, the energy efficiency of the LABs using G3 liquid electrolyte gradually decreased to 45% at the 100^th^ cycle. On the contrary, the LABs using DAP additive in G3 electrolyte shows an energy efficiency up to 80% at the beginning, and still achieves 60% after 250^th^ cycle. This means the well catalytic effects of DAP at the cathode. It is more clearly that the charging potential of LABs with DAP is significantly lower than that of LABs with pure liquid electrolyte at the beginning of the cycle (Figure 3b,c). Specifically, the overpotential in the first cycle is only 0.73 V (about half of G3 liquid electrolyte 1.3 V). After ≈ 25 cycles, it still kept ≈ 0.89 V.

In order to further illustrate the role of DAP additives in ether‐based solvents, the highest occupied molecular orbital (HOMO) of DAP, G3, LiTFSI were studied by density functional theory (DFT) calculation as shown in Figure 3d.^[^ ^17^ ^]^ The results show that the theoretical oxidation potential of DAP is much lower than that of G3 and LiTFSI. To verify the electrochemical activity of DAP were conducted on LABs with and without DAP under ambient air shown in Figure 3e. The reduction peaks of LABs with and without DAP are very similar, indicating that only slight influence of DAP on ORR. In sharp contrast, when the voltage exceeds 3.5 V, the current of LABs containing DAP additives significantly increases. Obviously, a lower onset potential and higher current density indicates that the DAP additive improves the kinetics of OER. The above results show that the oxidation potential of DAP is about 3.5 V, this means the charging platform of DAP as the RM catalyzed decomposition of discharge products is about 3.5 V. Moreover, highly active singlet oxygen (^1^O_2_, the first excited state of oxygen) which generated during discharging, has been proven to be a crucial factor in the occurrence of side reactions in LABs. It mainly experiences a disproportionation reaction of superoxide anions (O_2_^−^) during charging and discharging, as well as the oxidation reaction of superoxide or peroxide. The strong electron affinity of ^1^O_2_ can easily attack most of the electrolyte and electrode components, such as oxidizing organic solvents, passivating electrodes, etc., leading to capacity degradation and premature battery death. The effects of quenching ^1^O_2_ by DAP has also been investigated. We quantified the production of ^1^O_2_ by monitoring the concentration changes of 9,10‐dimethylanthracene (DMA) through UV–vis testing before and after charging and discharging (Figure S14, Supporting Information).^[^ ^18^ ^]^ The chemical probe DMA was added to two electrolytes respectively, and after cycling, the residual electrolyte was collected from the glass fiber separator for UV–vis test. As shown in Figure 3f, after the introduction of DAP, the DMA content in the electrolyte was higher compared to the electrolyte without DAP, indicating that the electrolyte without DAP produced more highly active ^1^O_2_. That is, the introduction of DAP could inhibit the production of ^1^O_2_. This can be attributed to the quenching effect of organic amines on singlet oxygen, and the decrease in charging overpotential assisted adding DAP, there was no significant change in the charging overpotential except for a slight DAP itself could also suppress the generation of singlet oxygen. The DAP additive also serves a function in LABs constructed with the DMSO electrolyte, thereby validating the universality of the DAP additive in LABs (Figure S15, Supporting Information). Meanwhile, the Li‐CO_2_ batteries were assembled to further illustrate the effects of DAP. As shown in Figure S16 (Supporting Information), before and after adding DAP, difference in the first cycle. It means the DAP additive has merely catalytic effect on the by there was no significant change in the charging overpotential except for a slight difference in the first cycle. It means the DAP additive has merely catalytic effect on the decomposition of Li_2_CO_3_. This indicates that the increase in overpotential in the later stage of LABs circulation may be caused by the accumulation of Li_2_CO_3_ with prolonged cycles.

**Figure 4:**
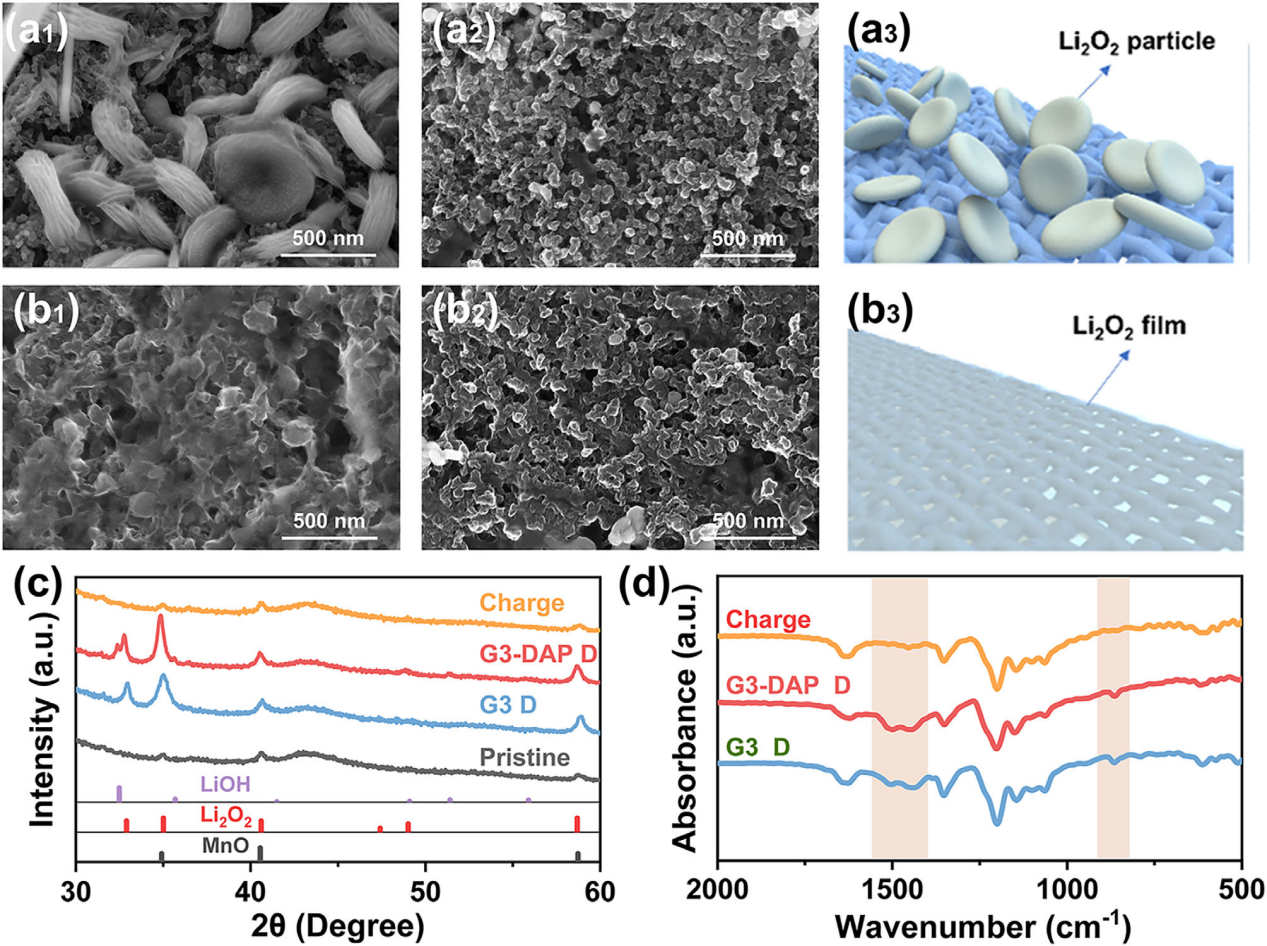
Characterizations the reversible formation/decomposition of discharged products. Morphology of discharged and charged cathodes with (a) G3 and (b) G3‐DAP electrolyte. c) XRD patterns and (d) FTIR spectra of discharge products at different electrolyte.

To further exploring the battery reaction mechanism and reversibility, multiple techniques were adopted to analyze the discharge products. The LABs are subjected to galvanostatic discharge at 250 mA g^−1^ for 20 h first, and then the cathode was collected for characterizations. The morphology of the cathode was observed by using scanning electron microscopy (SEM). **Figure** **4**a,b, shows the surface morphology of cathode after discharge and charge for a certain period of time. We found that with original G3 liquid electrolyte, a circular cake‐shaped particles (Figure 4a_3_) were deposited on the electrode surface, which had a similar morphology to the common Li_2_O_2_ particles. They were uniformly distributed on the electrode surface. The granular particles show poor contact with the cathode interface, which would hinder the transfer of ions during the charging process. After introduction of DAP additive, a thin film (Figure 4b) uniformly distributed on the electrode surface, an excellent contact between Li_2_O_2_ and the electrode, ensuring the rapid transmission of Li^+^. The main discharge product was determined to be Li_2_O_2_ by X‐ray diffraction (XRD) (Figure 4c), which can be effectively decomposed during the subsequent charging process. In addition, there is a peak near 875 cm^−1^ in the infrared spectrum (Figure 4d) and two peaks around 1400–1550 cm^−1^, belonging to Li_2_CO_3_, which indicating the production of Li_2_CO_3_ with low crystallinity.^[^ ^19^ ^]^ It is associated with the side reactions between Li_2_O_2_ and CO_2_ in the ambient air, as well as carbon corrosion in the electrodes. These results indicate that the discharge products include Li_2_O_2_, Li_2_CO_3_ and show high reversibility during discharging/charging cycles.

The gas permeability experiments were carried out on the G3 liquid and G3‐DAP gradient gel electrolyte to further disclose the Li anode protection mechanism. The gradually Li corrosion would deteriorate the storage performance of LABs and lead to poor calendar life.

**Figure 5:**
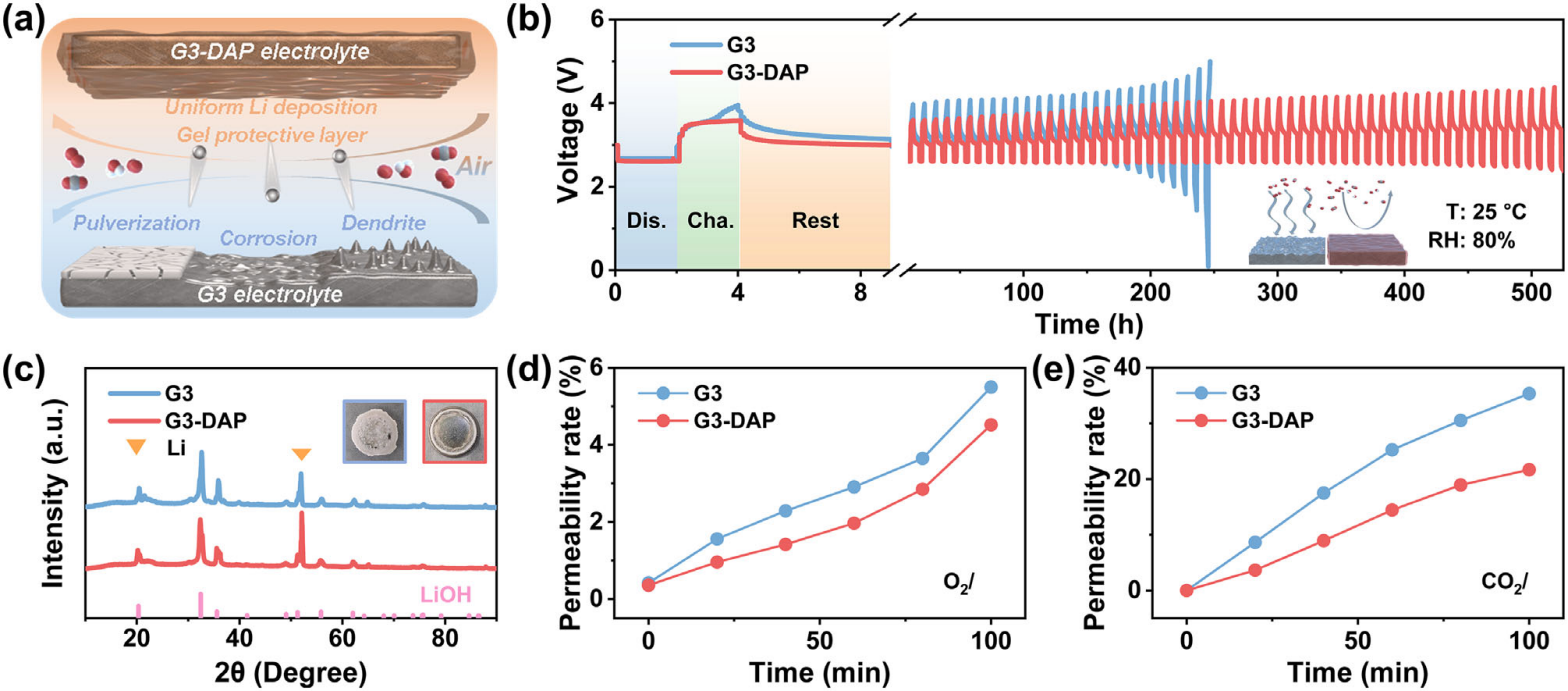
The inhibition of gas‐permeability through electrolyte by DAP additive. a) Schematic diagram of gel blocking air corrosion of Li anode. b) Calendar aging performance of LABs with G3 and G3‐DAP electrolyte in high humidity ambient air. c) The XRD patterns on the surface of the Li anode and the corresponding photograph. d) The permeability rate of O_2_ and e) CO_2_ through two electrolytes determined by GC.

The LABs with different electrolyte were assembled and subjected to galvanostatic discharge/charge and following a rest at a relative humidity of 80%. The inhibition effect of gel electrolyte on the diffusion of water vapor was tested by using self‐made equipment at a high humidity environmental. **Figure** **5**a shows that the gel on the surface of the Li anode effectively blocks the corrosive gas in the air and thus inhibiting the corrosion of Li anode and the growth of dendrites during the cycle. Figure 5b displays a single cycle sequence of LABs, that is, after one cycle of discharge and charge, they rest at open circuit voltage for 5 h, and then carry out the next cycle. The voltage profiles show that with DAP additive in the electrolyte (Figure S17, Supporting Information), the LABs can stably cycle for more than 500 h, which is much higher than G3 liquid electrolyte. During the long‐time rest, the moisture in high humidity would diffuse across the electrolyte and access the Li anode. Severely corrosion would be occurred and limited LABs cycling life. The XRD patterns on the surface of the Li anode and the photos of metallic Li anode after exposing to high humidity for 200 h (Figure 5c), proved that the addition of DAP result in the formation of gel electrolyte at the surface of Li and its corrosion by moisture has been greatly prohibited. To further investigate the gas/moisture proof capability of as‐formed gel electrolyte, gas chromatography was used to detect the permeability of O_2_ and CO_2_. With a home‐made equipment, the gas samples were taken every 20 min. As shown in Figure 5d,e, the diffusion of O_2_ and CO_2_ in gel and liquid electrolyte was similar, which the gas diffusion in gel electrolyte was great suppressed. The above results prove that the gel electrolyte layer can effectively block the corrosion of the Li anode, thus achieving the long‐term cycling of the LABs in ambient air conditions.

## 3 Conclusion

In summary, we have developed a bi‐functional electrolyte additive, DAP, for LABs. By introducing organic amine DAP additive into the G3 electrolyte, on the Li anode side, DAP spontaneously reacts with Li metal to form a Li‐DAP layer on the surface, and then in situ cross‐link with G3 molecule to form gel layer, which blocks the penetration of H_2_O, O_2_ and CO_2_ from ambient air, and retards the corrosion of Li metal. The high Li^+^ transference number of gel electrolyte also helps to inhibit the generation of Li dendrites on the Li anode surface. Meanwhile, the DAP remains liquid on the air cathode and it acts as redox mediator, improving the reaction kinetics of OER and energy efficiency of LABs. At the same time, it has a certain inhibitory effect on the production of ^1^O_2_. As a result, the LABs with DAP additive in electrolyte achieves a cycle life of over 1000 h and still maintains 60% energy efficiency after 250 cycles in ambient air. This work highlights the critical role of electrolyte additive to simultaneously protect the Li anode and serve as redox mediator at cathode to decrease the charging overpotential. The findings provide valuable insights for the development of practical, ambient‐operable LABs, marking a significant advancement in the field of metal‐air battery technology.

## Conflict of Interest

The authors declare no conflict of interest.

## Supporting information



Supporting Information

## Data Availability

The data that support the findings of this study are available from the corresponding author upon reasonable request.
