# Supplementary material for: Bi‐Functional Diaminopropane Additive Enables Stable Li Anodes and Highly Efficient Cathodes for High‐Performance Li‐Air Batteries
Source: Adv Sci (Weinh). 2025 May 29;12(32):e05539. doi: 10.1002/advs.202505539 (PMC12407377; doi:10.1002/advs.202505539)
Supplement: Supplementary file 1 — Supporting Information [file ADVS-12-e05539-s001.docx]

Supporting Information

Bi-functional Diaminopropane Additive Enables Stable Li Anodes and Highly Efficient Cathodes for High-performance Li-air Batteries

*Honghao Hu^a,b #^, Qingxu Zhang ^a,b #^, Jiucong Liu ^a^, Junjie Li ^b^, Pingli Wu^a^, Caicai Li^a^, Peipei Du^a^, Huiqiao Li^a,c^ and Xizheng Liu ^a,b^**

^a^Key Laboratory of Flexible Optoelectronic Materials and Technology, Ministry of Education, School of Optoelectronic Materials & Technology, Jianghan University, Wuhan 430056, China.

^b^Institute for New Energy Materials and Low-Carbon Technologies, School of Materials Science and Engineering, Tianjin University of Technology, Tianjin 300384, China

^c^State Key Laboratory of Materials Processing and Die & Mould Technology, School of Materials Science and Engineering, Huazhong University of Science and Technology, Wuhan 430074, China

^#^These authors contributed equally to this work.

*****Corresponding authors**

E-mail: [xzliu@jhun.edu.cn](mailto:xzliu@jhun.edu.cn)

**Experimental Section**

**Chemical and Materials:**

All of the reagents including Tetraethylene glycol dimethyl ether (G4, 99%), triethylene glycol dimethyl ether (G3, 99%, 0.01% BHT), diethylene glycol dimethyl ether (G2, 99.5%), Ethylenediamine (EDA, 99.5%), 1,3-Propanediamine (DAP,98%), Diethylenetriamine (DETA, 99%) and Lithium bis(trifluoromethanesulphonyl)-imide (LiTFSI, ≥99.9%) were purchased from Aladdin Reagent, Lithium sheets (15.6 Dia×0.65 mm T, 99.9%) was obtained from Canrd. All of the chemical were used as obtained without any additional purification.

**Synthesis of the porous MnO nanoflower**

The porous MnO nanoflowers were synthesized according to our previous work with some modifications. ^[1]^ Firstly, the Mn/Al alloy foils were prepared by pure Mn and Al (99.9 wt.%) in a 5:95 atomic ratio by single roller melt spinning method. Then, 100 mL NaOH (2.0 M) was added dropwise into a plastic beaker with a mixture of 0.2 g alloy foils, 72 mL H_2_O, and 28 mL H_2_O_2_(30 wt.%). Under the condition of stirring at room temperature and keeping for 8 h, the products (Na_0.55_Mn_2_O_4_) were collected and dried at 60 °C in a vacuum oven overnight. Subsequently, the as-prepared Na_0.55_Mn_2_O_4_ was annealed at 500 °C for 2 h in an air atmosphere at an increasing rate of 1 °C min^-1^, followed by 5 h in Ar/H_2_ atmosphere at 350 °C at the same heating rate. The final product porous MnO nanoflowers were washed several times with deionized water and dried in a vacuum oven at 60 °C to remove the water.

**Synthesis of the ex-situ G3-DAP gel**

2 pieces of Lithium sheets with a diameter of 15.6 mm and thickness about 0.65 mm were immersed in 1 mL G3 solution with 20% DAP (volume ratio) for one day. Then, the Li sheets were separated and stored the residual solution at room temperature. The G3-DAP gelation gradually occurred within two days. All of the experiments were completely in an argon-filled glove box. Other kinds of ether-amine-based gels were obtained with the same methods, including replacing G3 by G2 and G4, and replacing DAP by EDA and DETA.

**Materials characterization**

The X-ray diffraction (XRD) patterns were collected using a MiniFlex600 diffractometer (Rigaku, Japan) with Cu radiation (Cu Ka 0.15406 nm). Scanning electron microscope (SEM) with FEG was conducted using a Verios 460L microscope (FEI). Fourier transform infrared spectroscopy (FT-IR) tests were performed on a Frontier Mid-IR FTIR spectrometer (PerkinElmer). Arbin multichannel electrochemical testing system (BT2043) was used to measure galvanostatic discharge/charge performance. Gas chromatograph (GC9790II, FULI INSTRUMENTS) was used to the gas permeability experiments which are carried out on G3-DAP gel and G3 liquid electrolyte. The linear sweep voltammograms and electrochemical impedance spectroscopy were performed on electrochemical workstation (CHI 760E).

**Li-air battery assembly**

The active materials for air cathode prepared by mixing Kejten black (KB), MnO, and Polytetrafluoroethylene (PTFE) at a weight ratio of 4.5: 4.5: 1, rolling into a film and coated on a carbon cloth current collector. The total areal loading of the composite of porous MnO and KB is about 1.0-1.5 mg cm^-2^. The air cathode and Li anode were separated by a Whatman glass fiber (GF/A) separator which was immersed by electrolyte (0.5M LiTFSI/G3 with and without DAP additive) sealed into coin-type CR 2032 with air holes. The G3-DAP electrolyte gelation gradually occurred after battery rest.

**Electrochemical measurements**

The galvanostatic discharge/charge performance were tested on Lanhe electrochemical system at a current density of 250 mA g^-1^ with a limited capacity of 500 mAh g^-1^ within the potential window between 2.0 and 4.5 V. The current density and specific capacity were calculated according to the total mass of MnO and KB. Electrochemical impedance spectroscopy (EIS) was conducted by using an electrochemical workstation (CHI 760E) at the frequency range from 0.01 Hz to 10 kHz. Similarly, the electrochemical stability of G3 liquid and G3-DAP gel electrolytes were investigated by the linear sweep voltammograms on electrochemical workstation (CHI 760E) at a scanning rate of 1.0 mV s^-1^. In order to determine the Li^+^ transference number ($t_{{Li}^{+}}$) of the electrolyte, a chronoamperometry method was employed to measure Li symmetric cells with different electrolytes. A constant polarization of 10 mV was applied to the cells for 6000 s. At the same time, the AC impedance spectra before and after the polarization were recorded under an amplitude of 10 mV with the frequency ranging from 0.01Hz to 10 kHz. The $t_{{Li}^{+}}$ was calculated with the following equation:

$$t_{{Li}^{+}}=\frac{I_{ss}\left( \Delta V-I_{0}R_{0} \right)}{I_{0}\left( \Delta V-I_{ss}R_{ss} \right)}$$

where ΔV is the polarization voltage applied, I_0_ and R_0_ refer to the initial current and resistance, respectively, and I_ss_ and R_ss_ represent the steady-state current and resistance, respectively. All electrochemical measurements of these LABs were carried out in ambient air with a relative humidity (RH) of 10~40%.

**Theoretical calculation methods**

The theoretical calculations were performed via the Gaussian 16 programs based on density functional theory (DFT). The structures of the studied molecules were fully optimized using the B3LYP functional with DFT-D3BJ dispersion correction^[2-4]^ combined with 6-31g* basis set. The vibrational frequencies of the optimized structures were carried out at the same level. The structures were characterized as a local energy minimum on the potential energy surface by verifying that all the vibrational frequencies were real. And the Gibbs free energy is calculated using the Shermo program. The HOMO/LUMO orbital distribution is visualized by gaussview program.

**Ex-situ ^1^O_2_ detection**

LABs used 30 µL electrolyte and cycled for 5 cycles in ambient air. The electrolyte consisted of 4.8mM and 0.5M G3-LiTFSI, and add 8% (vol.) DAP additive to the experimental group. The cells were disassembled immediately in a glovebox after cycle, and the separator was extracted and soaked in 3 mL of Diethylene glycol dimethyl ether (G2, which not impurity peak influence in the DMA characteristic peak range). Therefore, the concentration of DMA extracted without cycling is 48 µM, and the absorbance of DMA is calculated using Lambert Beer’s rule to obtain the absorbance at 378.7 nm. Afterwards, the DMA was thoroughly dispersed in the solvent under ambient air conditions by ultrasound for 10 min. The obtained solution was centrifuged at 10000 rpm for 10 min, and supernatant was taken and measured by UV--vis in a quartz tube.


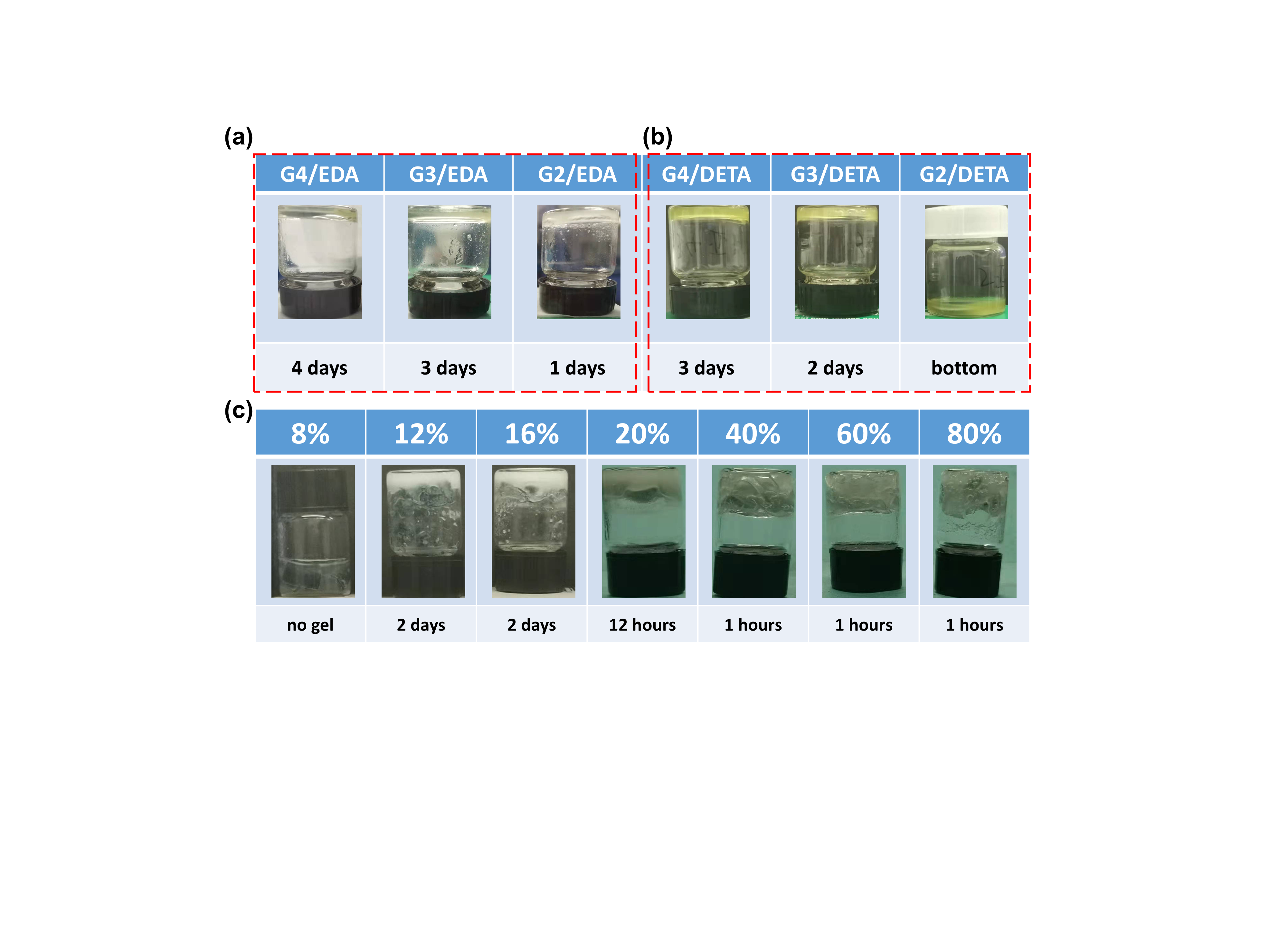


**Figure S1.** Optical photos of ex-situ gelation of different ether and amine components in Ar atmosphere. (a) G4, G3, G2 and 4% EDA ex-situ gelation experiments. (b) 4% DETA, and (c) Ex-situ gelation experiments of G3 and DAP with different ratios.


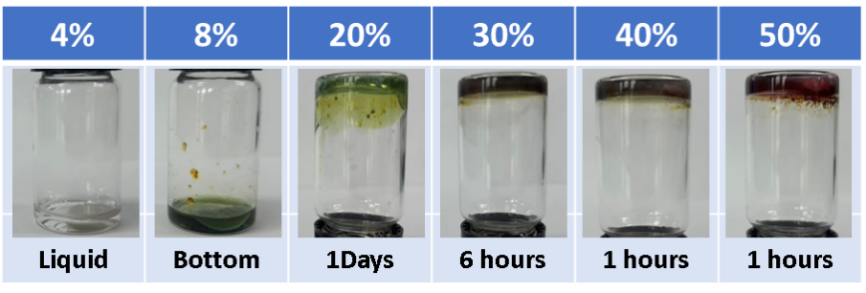


**Figure S2.** Optical photographs of reactions with different DAP content additives in G2 solvent.


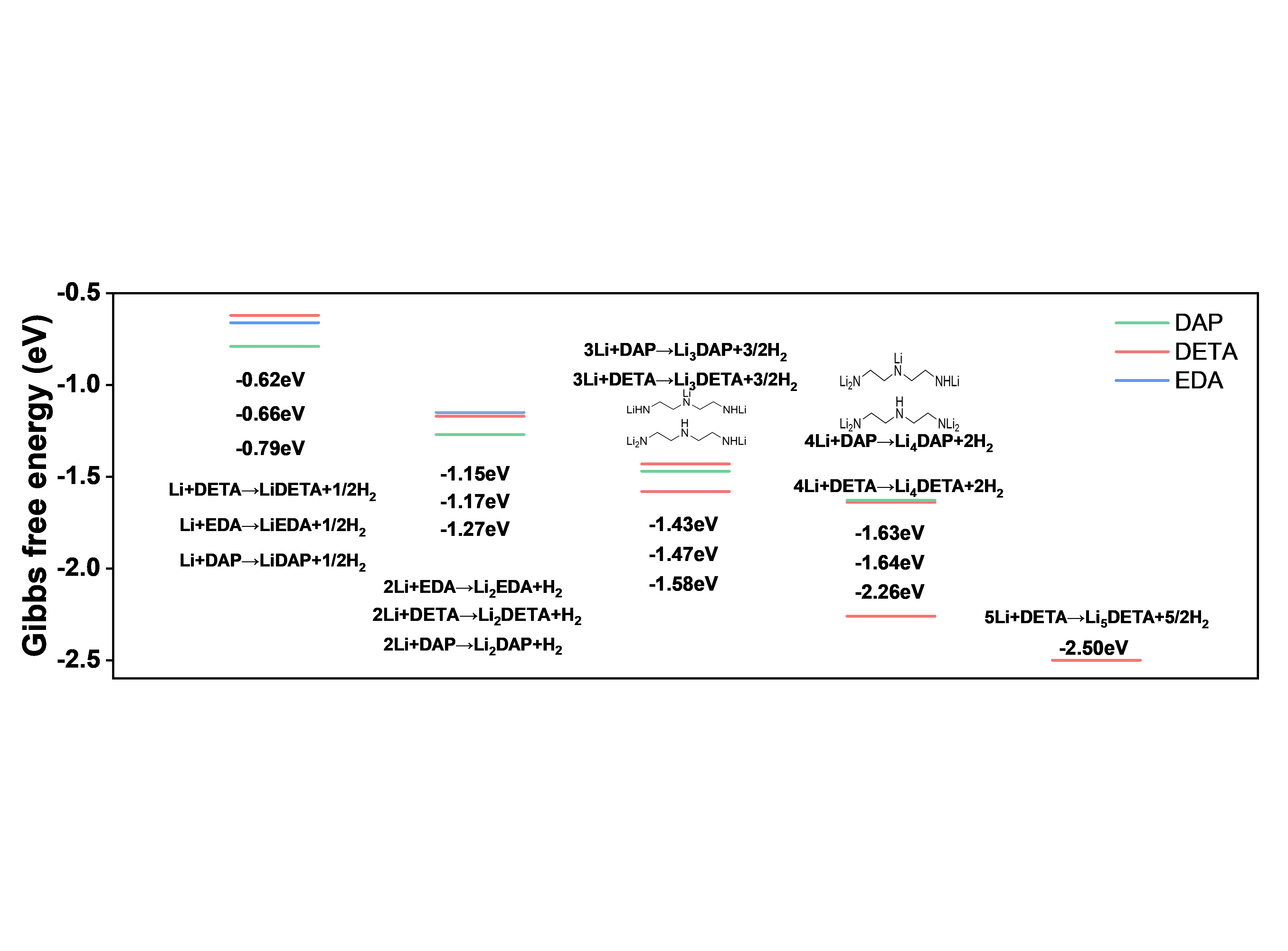


**Figure S3.** Gibbs free energy of possible chemical reactions between metallic Li and EDA, DAP, and DETA.


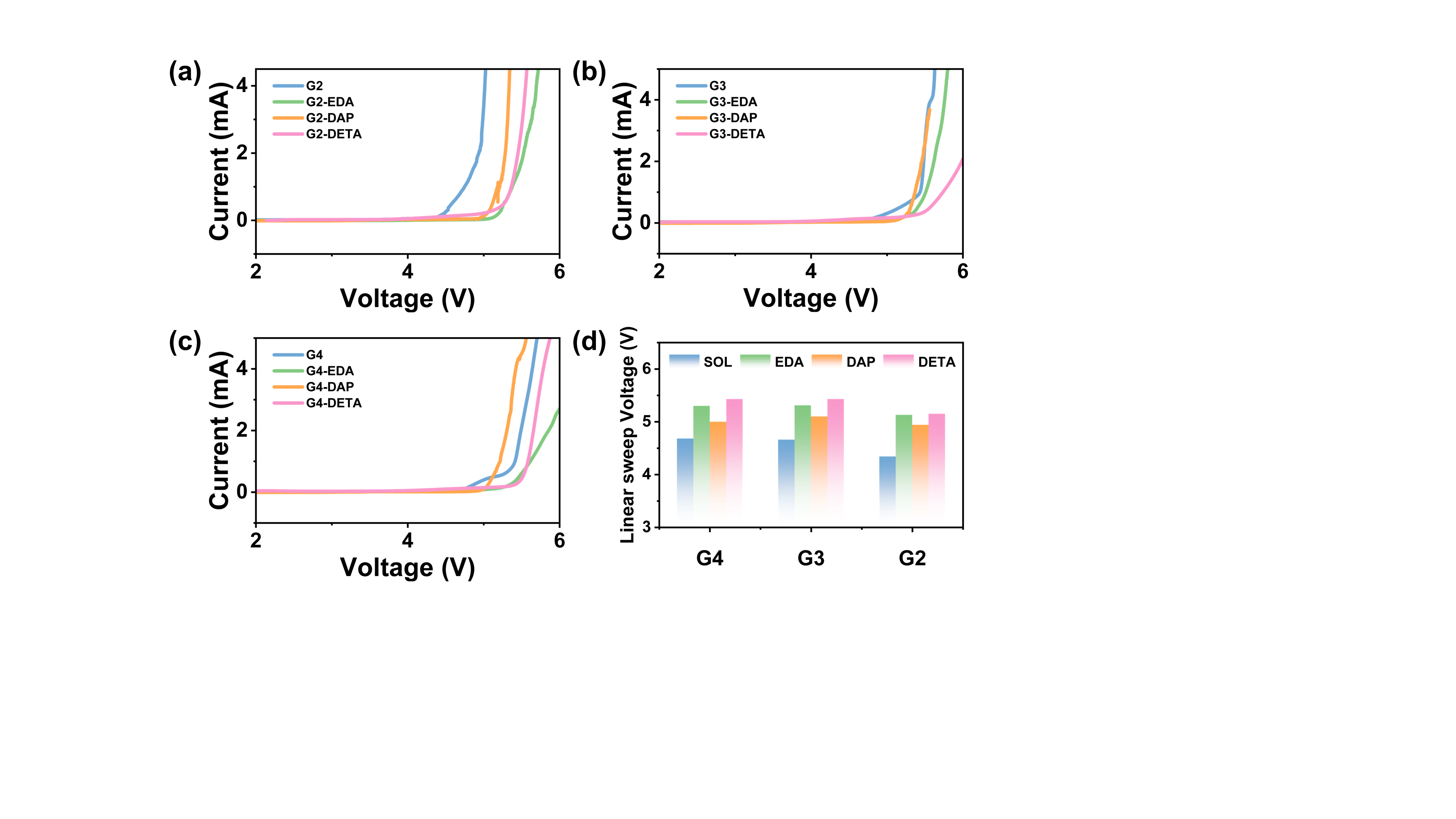


**Figure S4.** Electrochemical stability of different gel electrolytes. (a-c) Linear sweep voltammetry curve of different gel electrolytes and liquid electrolyte. (d) Summarization of onset potentials.


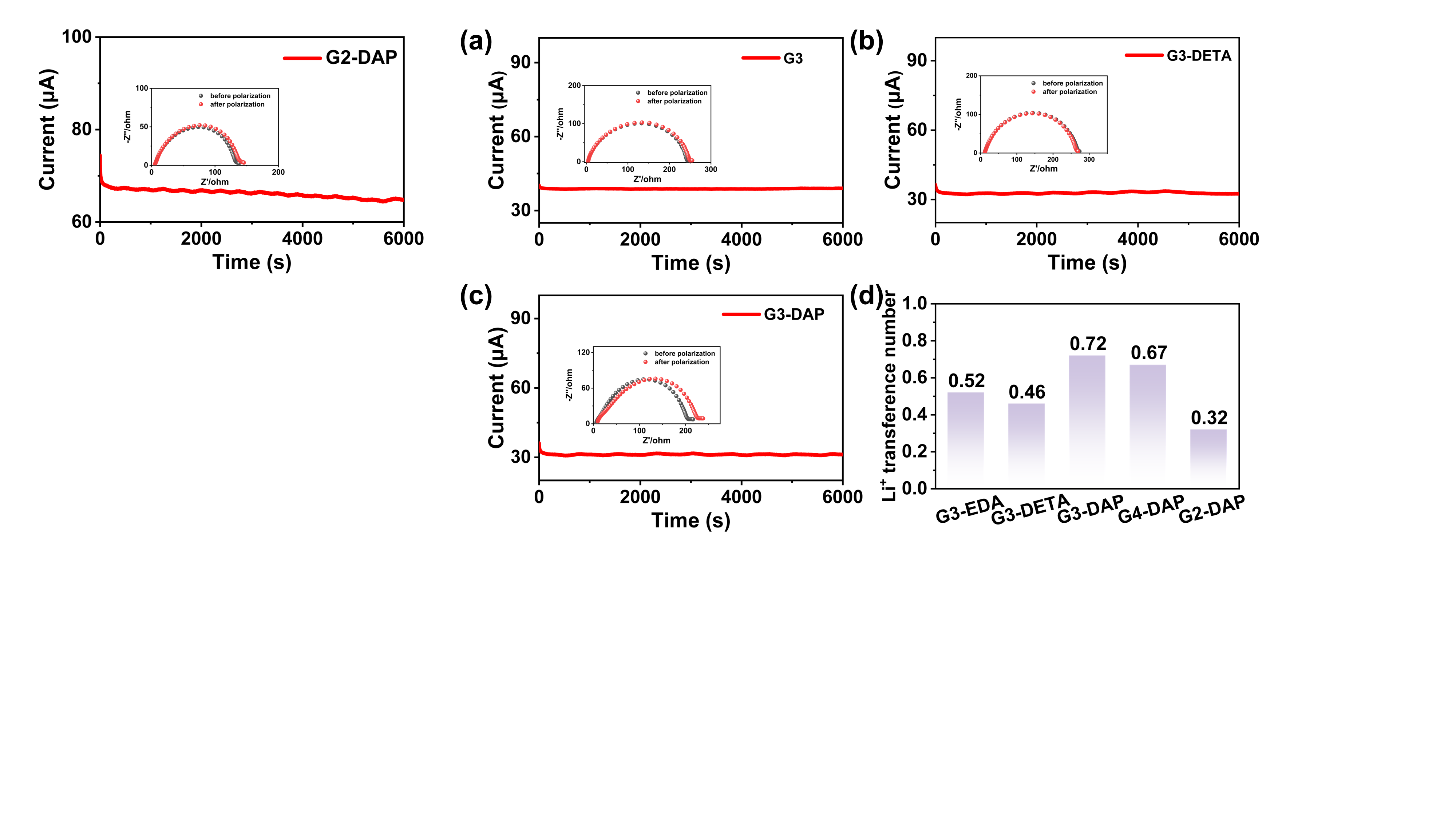


**Figure S5.** (a-c) DC polarization curves, Nyquist plots of initial and steady cells with G3, G3-EDA, G3-DAP, G3-DETA, electrolytes at 10 mV. (d) Summarization of Li^+^ transference number with different electrolytes.


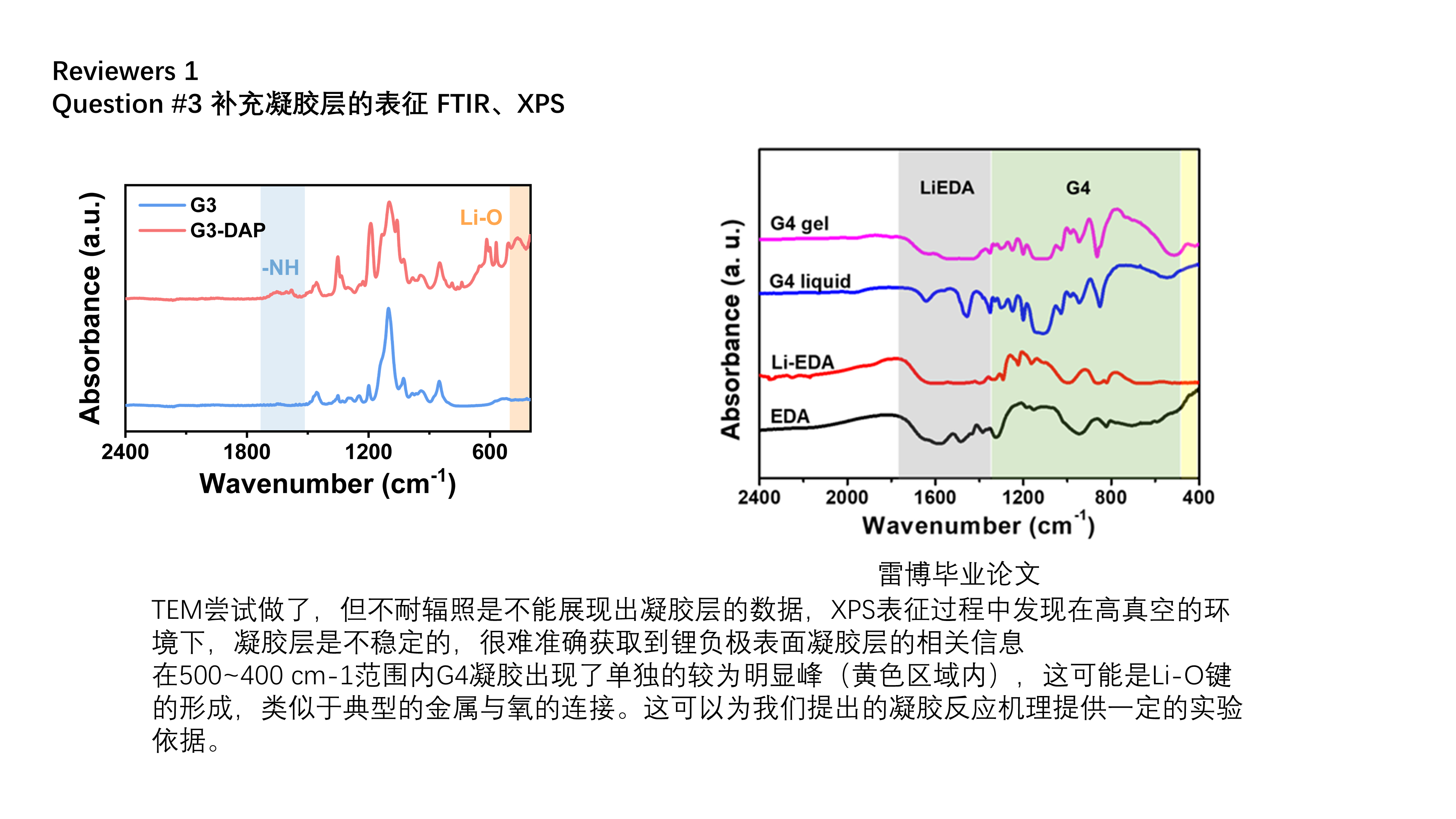


**Figure S6.** Fourier-transform infrared spectroscopy of the gel layer on the Li anode surface.


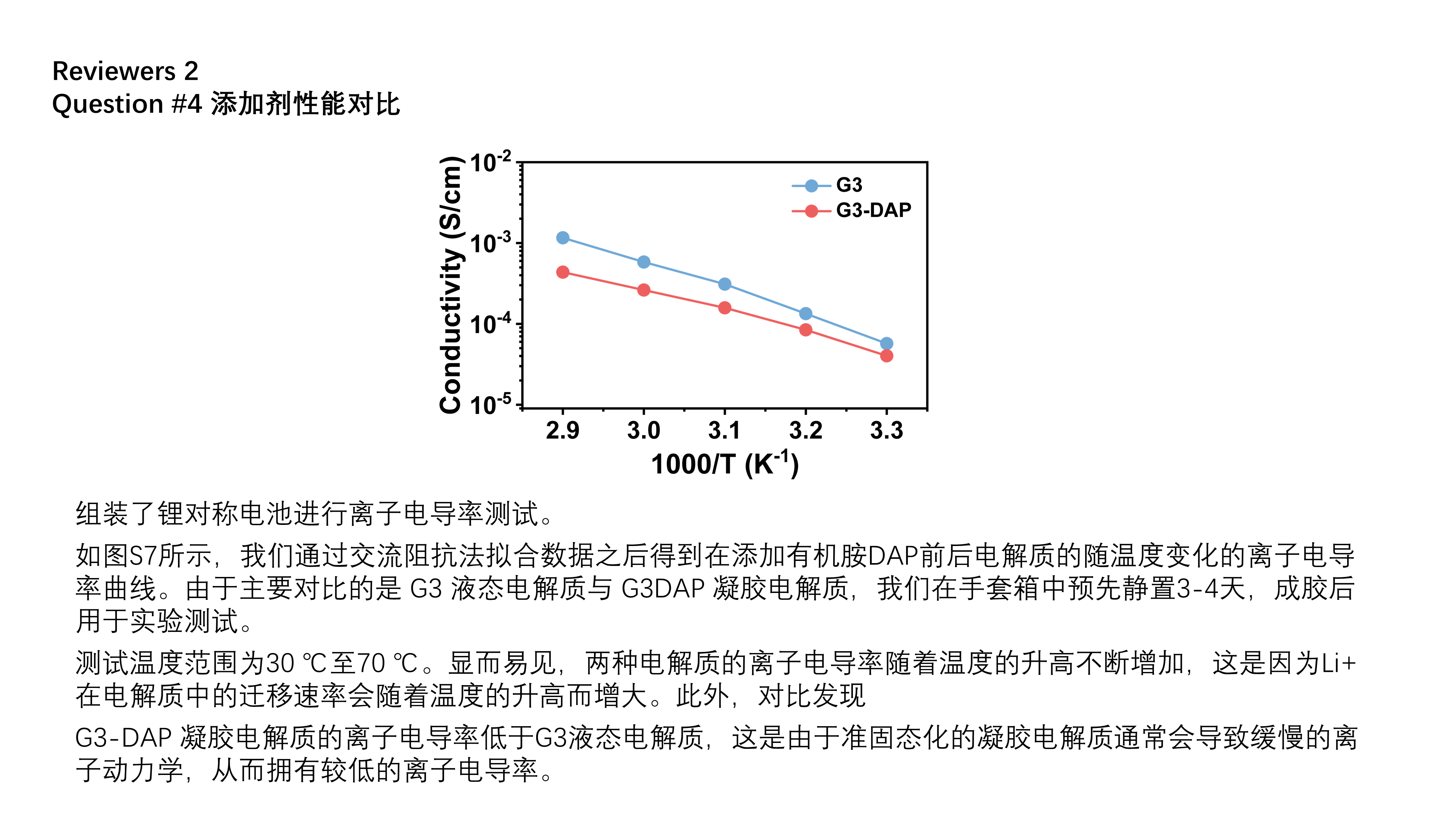


**Figure S7** Ionic conductivity of different electrolytes.


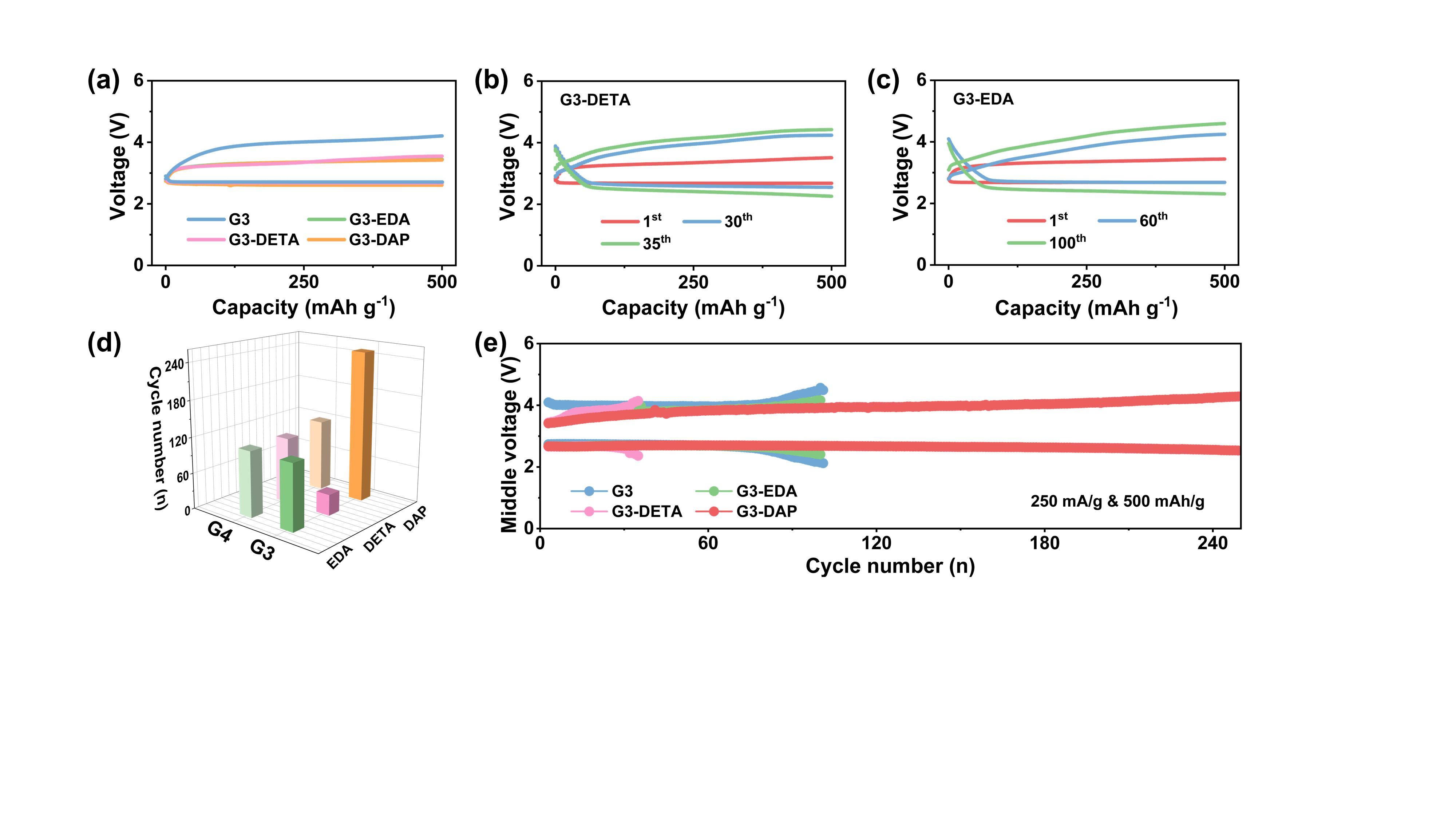


**Figure S8.** (a-c) The discharge/charge profiles of LABs with G3 liquid electrolyte and G3-EDA, G3-DETA electrolytes in ambient air. (b) Summarization of cycle number with G3 and G4 based electrolytes. (e) Cycling performance of LABs with different electrolytes.


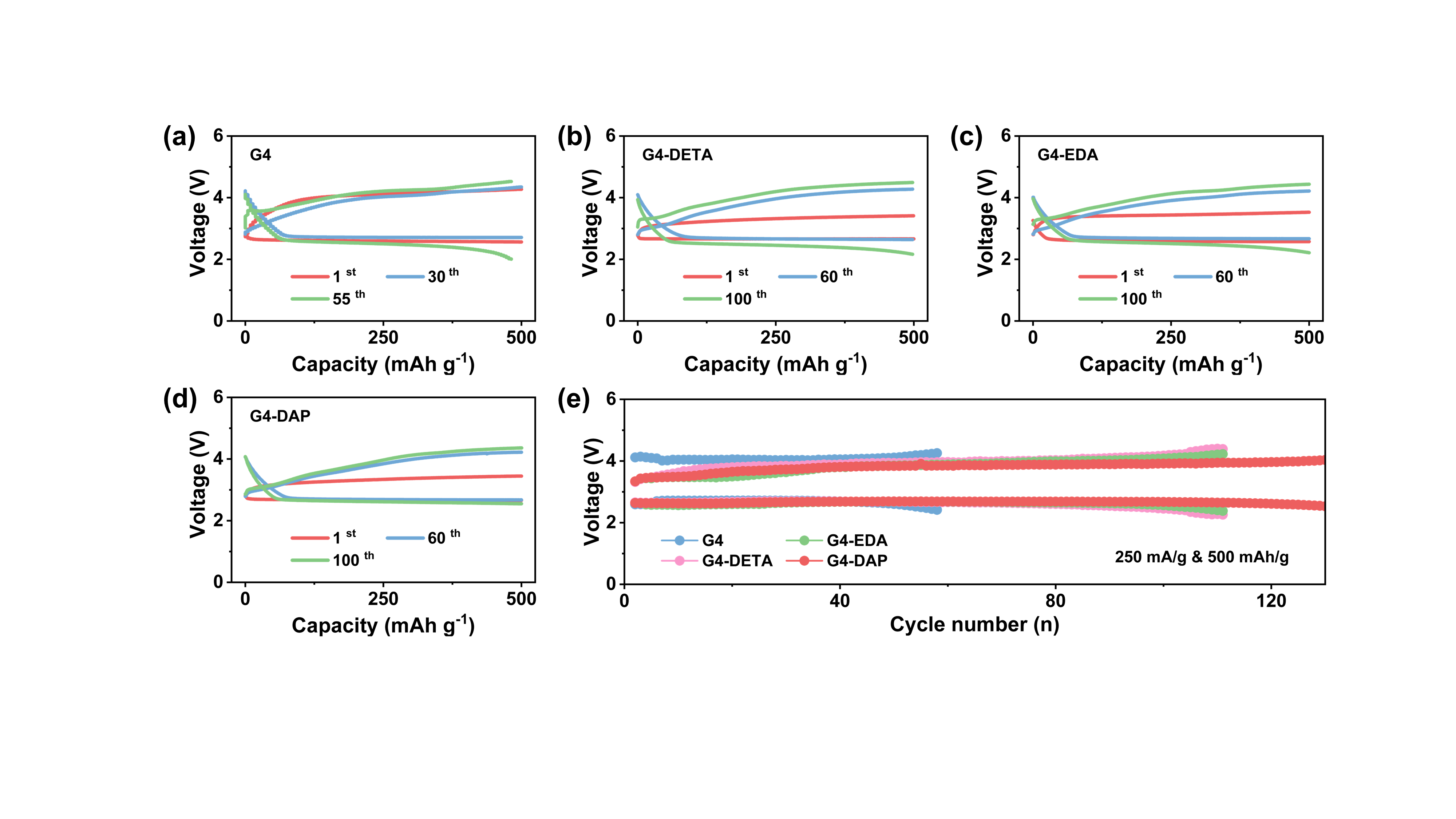


**Figure S9.** (a-d) The discharge/charge profiles of LABs with G4 liquid electrolyte and G4-EDA, G4-DETA, G4-DAP electrolytes in ambient air. (e) Cycling performance of LABs with G4-based electrolytes.


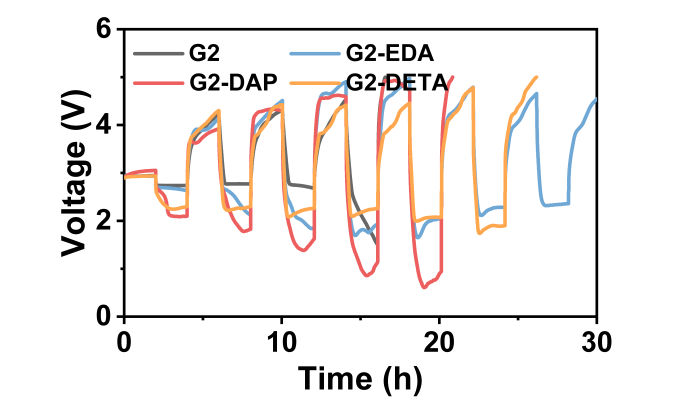


**Figure 10.** Cycling performance of LABs with different amine additives in G2 based electrolyte.


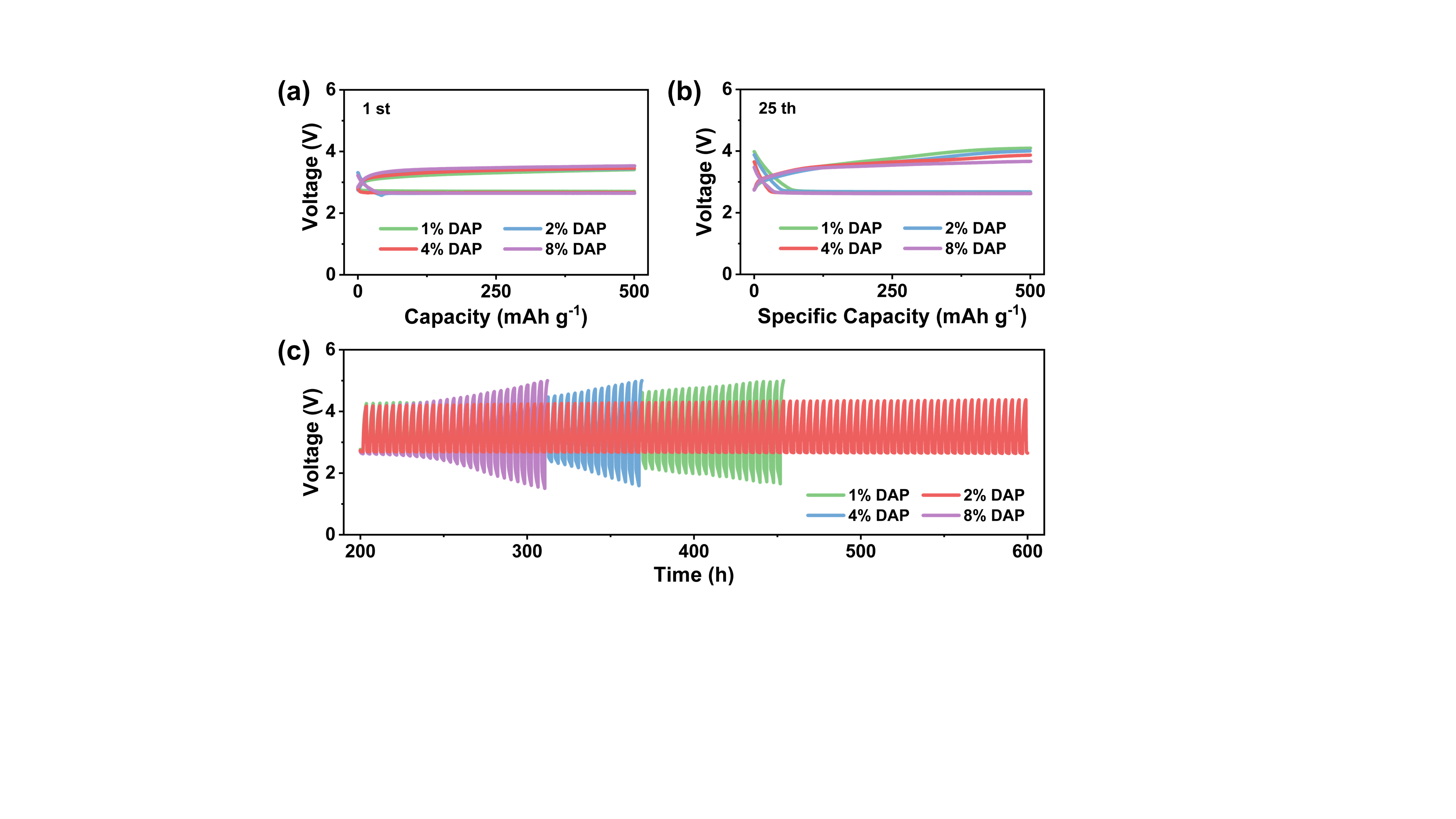


**Figure S11.** Cycling performance of LABs with 1%, 2%, 4%, and 8% of DAP additives at a current density of 250 mA g^-1^ and a limiting capacity of 500 mAh g^-1^ in ambient air. Specific capacity voltage profiles at the (a) 1^st^ and (b) 25^th^ cycles, respectively; (d) voltage profiles over 200-500 h.


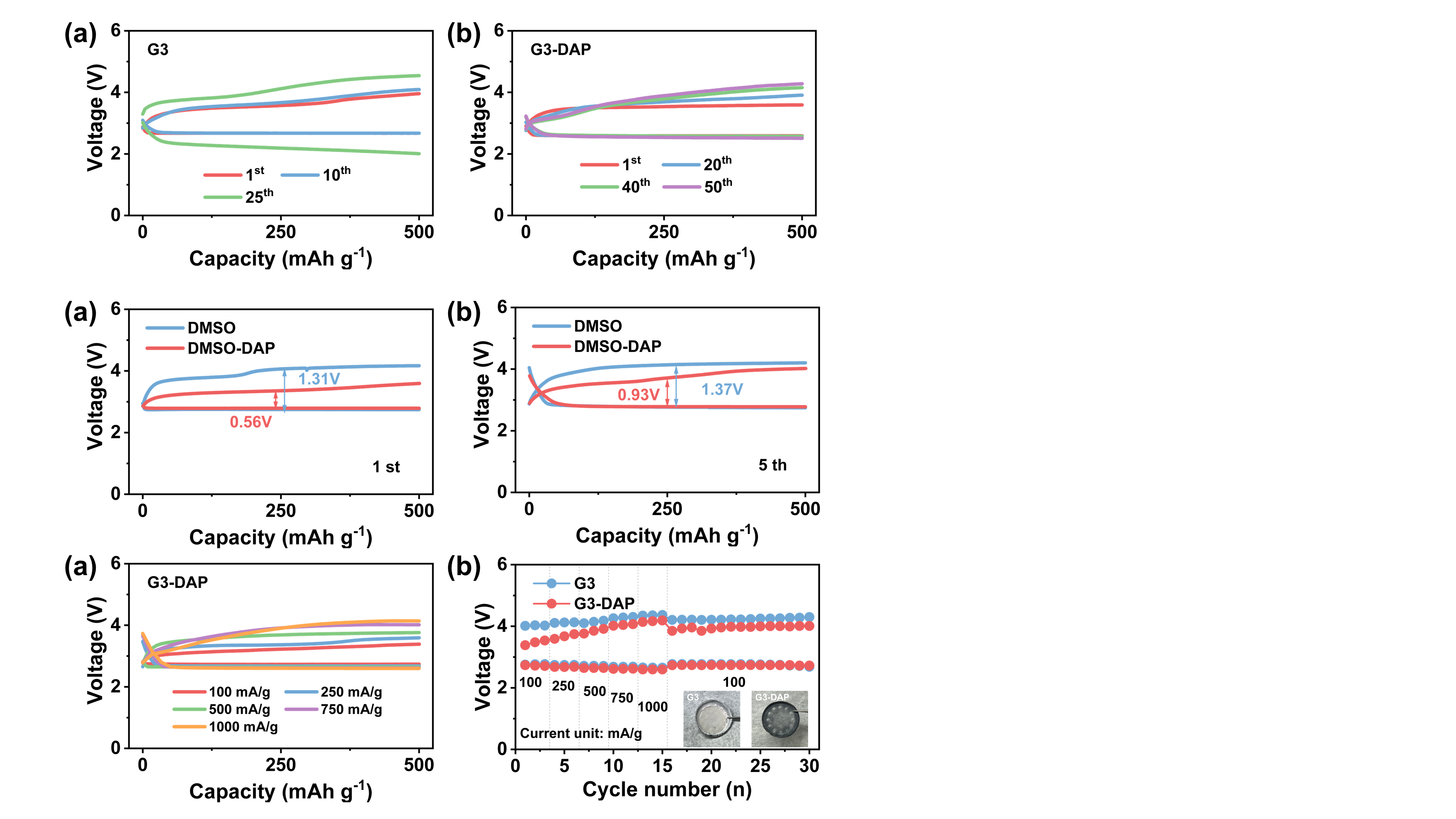


**Figure S12.** (a) Charge-discharge curves of LABs assembled with G3-DAP electrolyte at different current densities; (b) rate performance of different electrolytes (Inset: optical photographs of Li anodes after cycling).


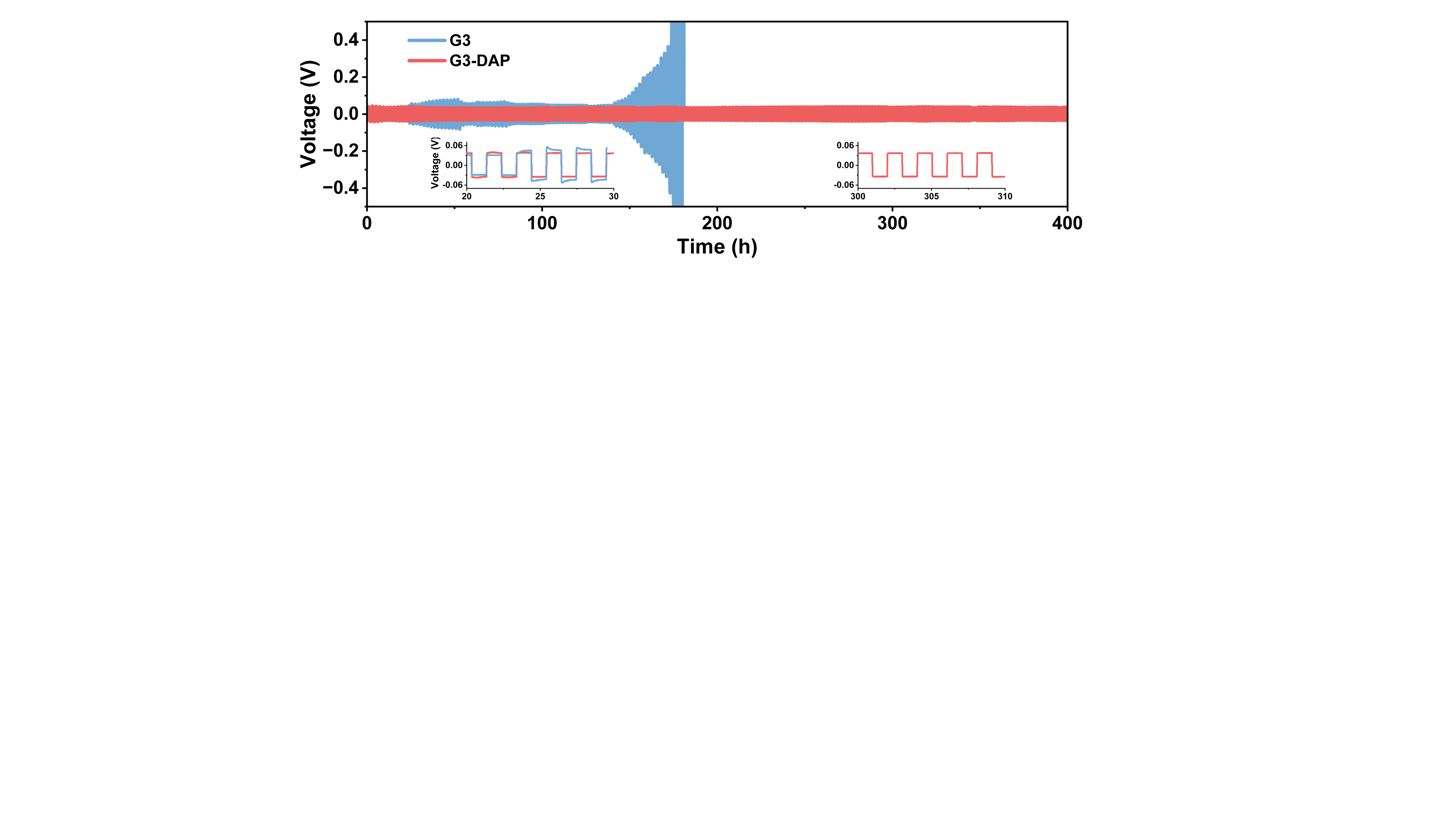


**Figure S13.** The reversible Li electroplating stripping curve of Li//Li battery with G3 liquid electrolyte and G3-DAP gel electrolyte at a current density of 0.1 mA cm^-2^ with a limited capacity of 0.1 mAh cm^-2^. Insets: enlarged profiles at the five consecutive cycle numbers noted.


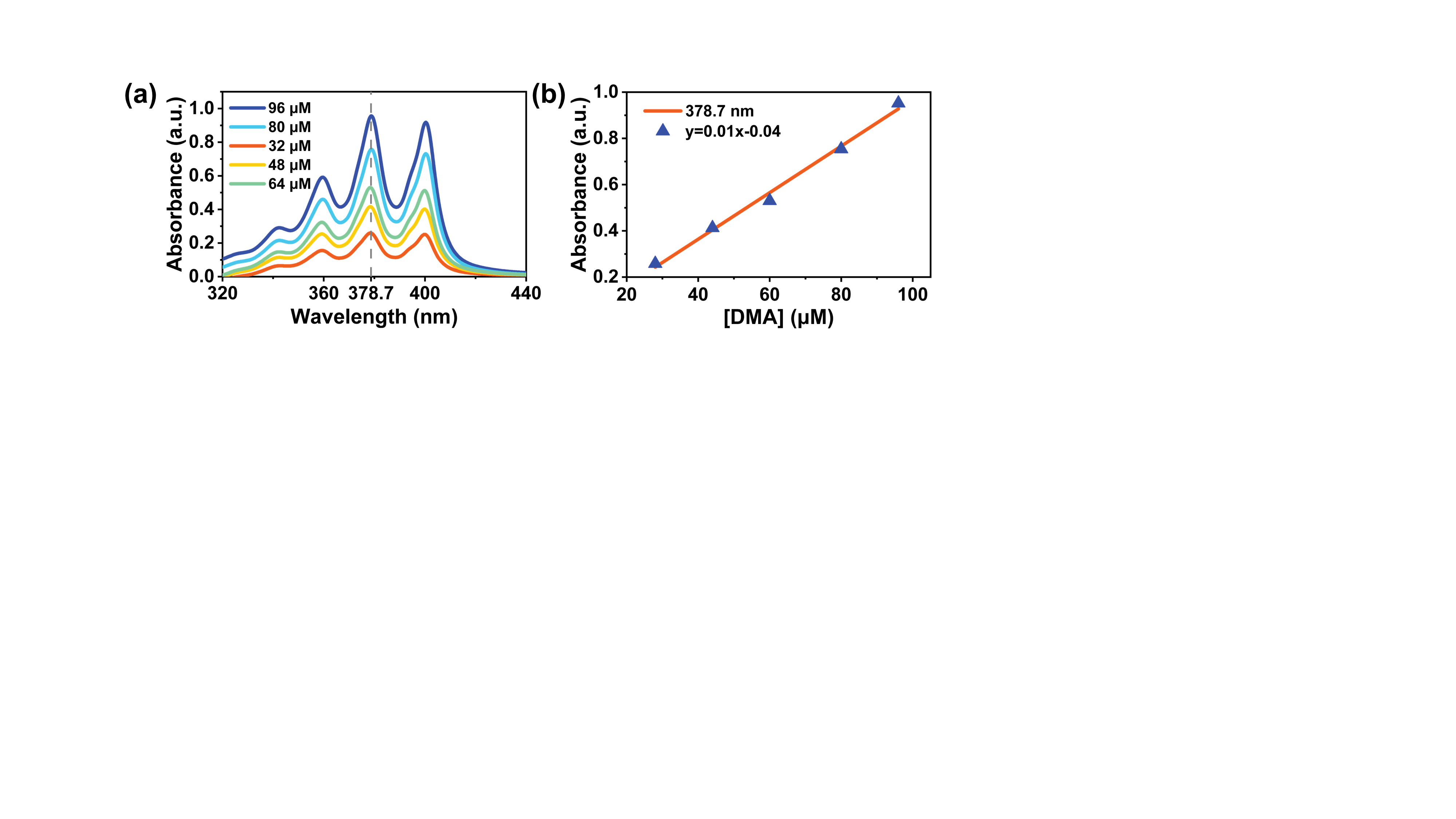


**Figure S14.** The detection of singlet ^1^O_2_. (a) UV absorption spectra of DMA solutions with different concentrations. (b) The fitting curve of the relative absorbance of DMA in G3 electrolyte at 378.7 nm with respect to the concentration dependence of DMA.


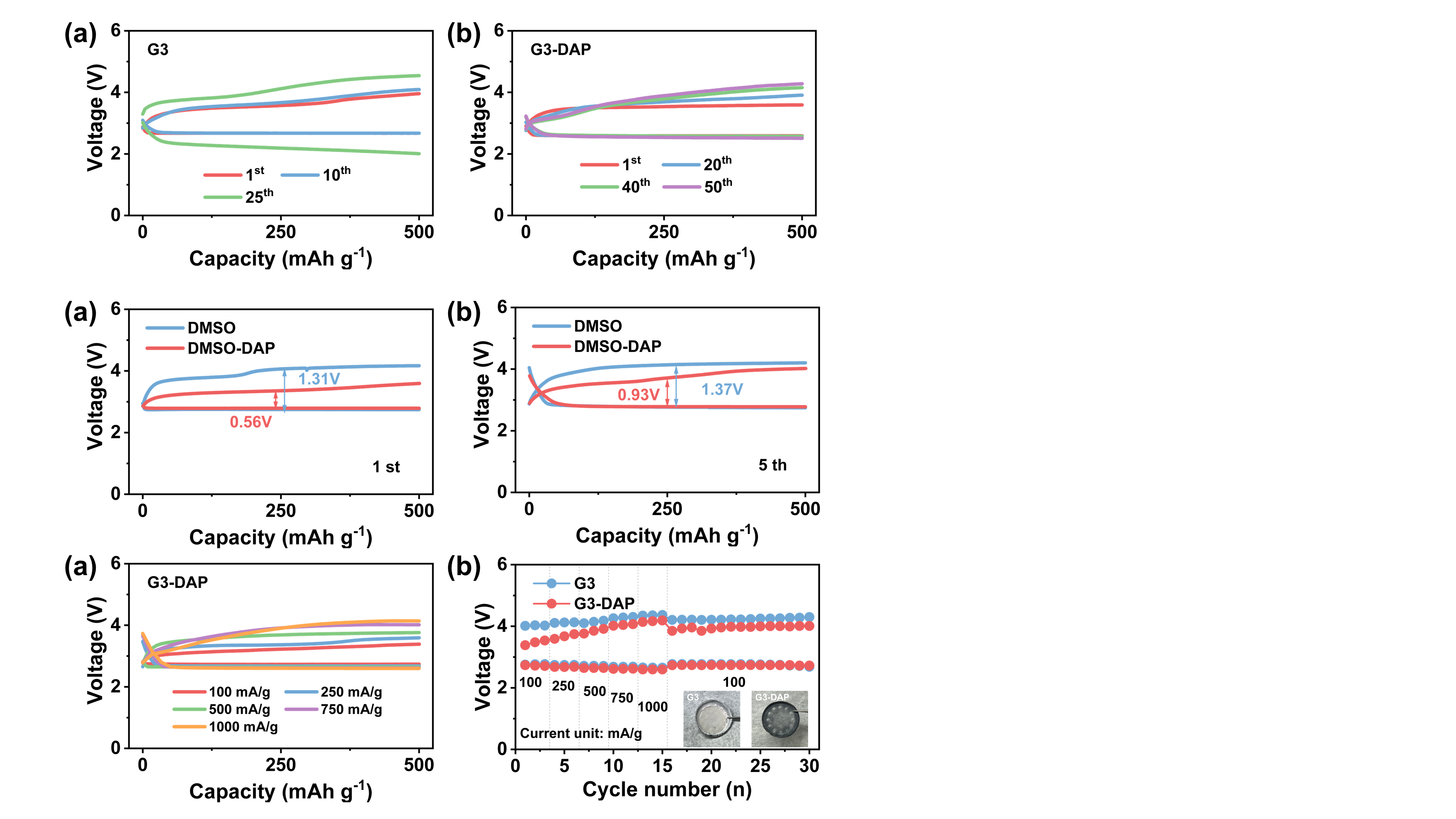


**Figure S15.** The discharge/charge profiles of Li-air batteries with (a) DMSO and (b) DMSO-DAP electrolytes.


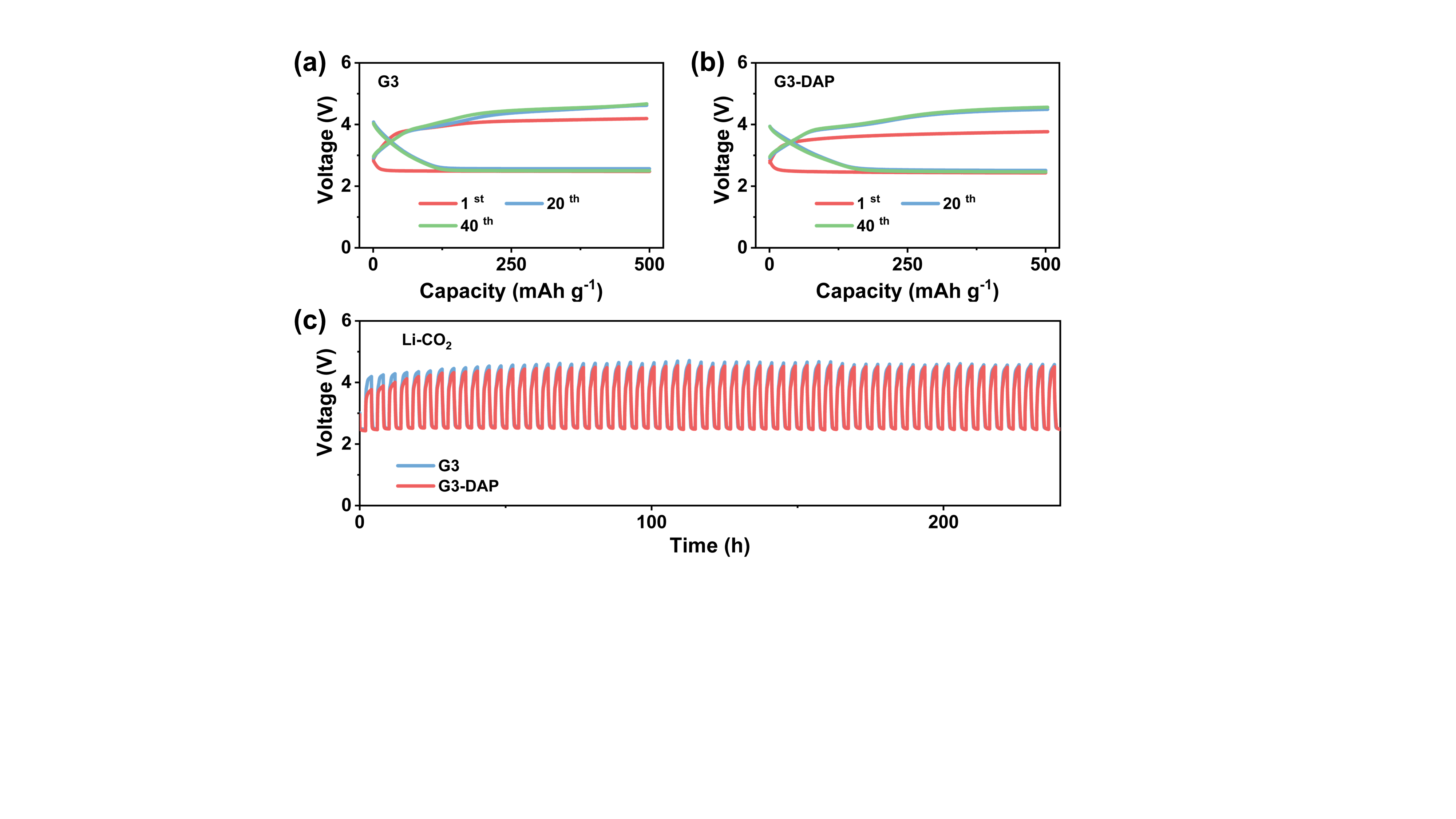


**Figure S16.** (a, b) The discharge/charge profiles of G3 and G3-DAP electrolyte in high humidity air environment. (c) Cycling performance of Li-CO_2_ batteries.


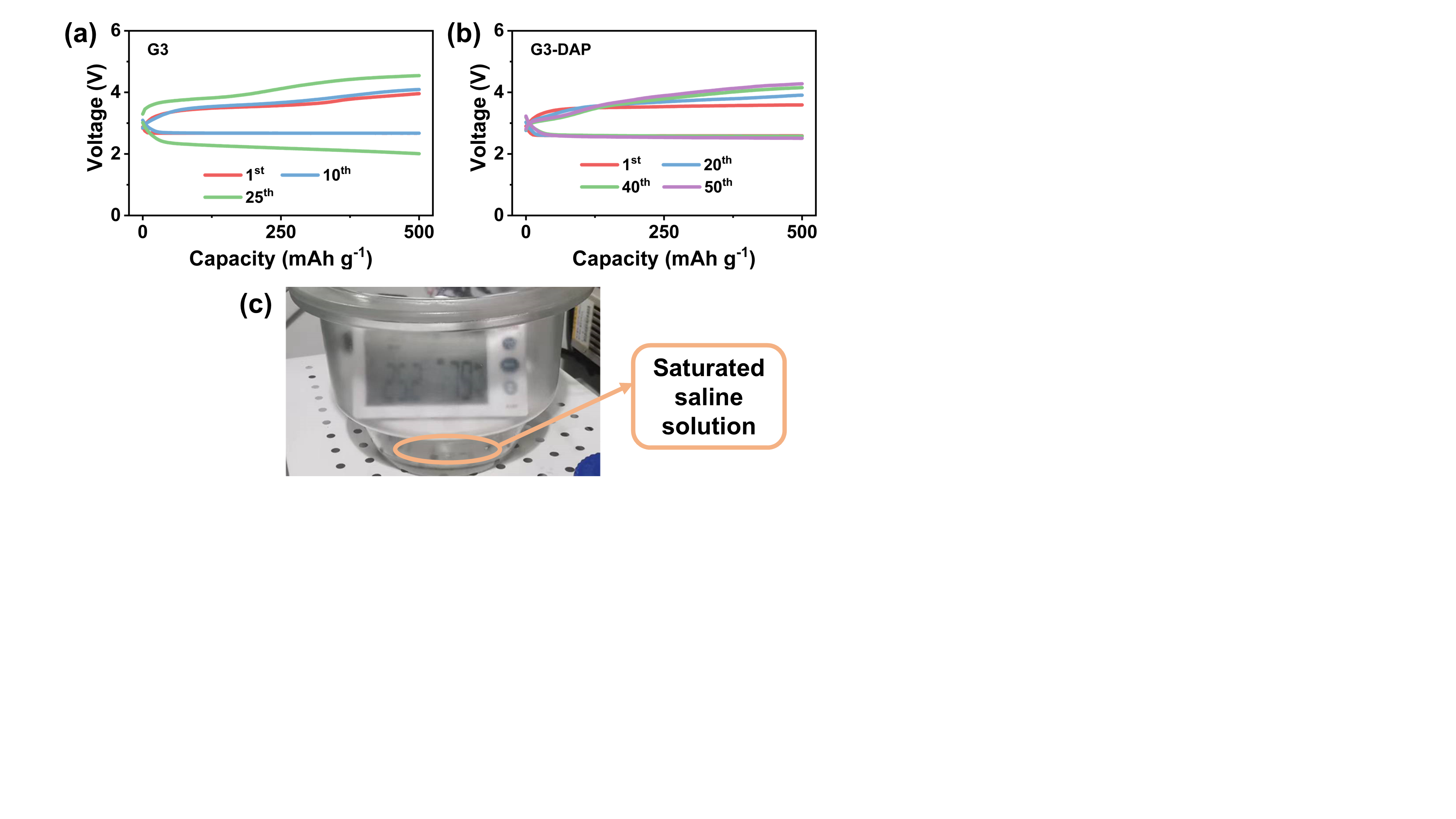


**Figure S17.** (a, b) The discharge/charge profiles of G3 and G3-DAP electrolyte in high humidity air environment. (c) Optical photo of home-made high humidity environmental equipment (Humidity can be controlled by using supersaturated solutions of different salts).

**Table S1**. Comparison of Li-air batteries perfoemance with different electrolyte additives.

| **Additive** | **Electrolyte** | **Cathode** | **Charge**  **overpotentials**  **(V)** | **Current density**  **(mA/g)** | **Limited capacity**  **(mAh/g)** | **Cycling time**  **(h)** | **Operating atomosphere** | **Reference** |
| --- | --- | --- | --- | --- | --- | --- | --- | --- |
| IMPBr | 1 M LiClO₄  DMSO | N-doped graphene | 0.74 | 400 | 500 | 112.5 | O_2_ | 5 |
| PTFEMA | 1 M LiTFSI TEGDME | GDL | ~0.9 | 500 | 1000 | 480 | O_2_ | 6 |
| B18C6 | 0.5 M LiTFSI  TEGDM | CNTs | 0.53 | 500 | 1000 | 1420 | O_2_ | 7 |
| RhB | 1 M LiTFSI  TEGDME | SP | ~0.8 | 500 | 1000 | 860 | O_2_ | 8 |
| PAMMA | 1 M LiTFSI  TEGDME | carbon cloth | ~0.9 | 500 | 1000 | 300 | O_2_ | 9 |
| DMII | 1 M LiTFSI  TEGDME | CB | 0.4 | 500 | 1000 | 960 | O_2_ | 10 |
| 15C5 | 1M LiNO_3_  DMSO | CNTs | 0.6 | 200 | 500 | 890 | O_2_ | 11 |
| DB24C8A | 0.5 M LiTFSI  TEGDME | CNTs | 0.77 | 500 | 1000 | 852 | O_2_ | 12 |
| DBDMB | 1 M LiCF_3_SO_3_  TEGDME | SP | 1.01 | 1000 | 1000 | 486 | O_2_ | 13 |
| EQ | 1 M LiTFSI  TEGDME | SP | 0.12 | 500 | 1000 | 600 | O_2_ | 14 |
| **DAP** | **0.5 M LiTFSI**  **TEGDME** | **MnO/KB** | **0.43** | **250** | **500** | **1000** | **Ambient air** | **This work** |

**Supplementary References**

[1] C. Yao, X. Lei, C. Ma, Q. Zhang, X. Liu, Y. Ding, *Small* **2023**, *19*, e2301846.

[2] T. Lu, Q. Chen, *Comput. Theor. Chem*. **2021**, *1200*, 113249.

[3] Z. Wang, W. Chen, T. Sang, Z. Zhang, M. Li, H. Tian, Z. Ming, *Surf. Interfaces* **2023**, *39*, 102893.

[4] J. Moellmann, S. Grimme, *J. Phys. Chem. C* **2014**, *118*, 7615.

[5] J. Lai, N. Chen, F. Zhang, B. Li, Y, Shang, L. Zhao, L. Li, F. Wu, R. Chen. *Energy Storage Mater.* **2022**, 49, 401-408.

[6] X. Wu, B. Niu, H. Zhang, Z. Li, H. Luo, Y. Tang, X. Yu, L. Huang, X. He, X. Wang, Y. Qiao, S-G. Sun. *Adv. Energy Mater.* **2023**, 13, 2203089.

[7] Q. Zhang, Y. Li, E. Poh, Z. Xing, M. Zhang, M. Wang, Z. Sun, J. Pan, S. Vummaleti, J. Zhang, W. Chen. *Adv. Energy Mater.* **2023**, 13, 2301748.

[8] H. Wan, Y. Sun, W. Cai, Q. Shi, Y. Zhu, Y. Qian. *Adv. Funct. Mater.* **2022**, 32, 2106984.

[9] X. Wu, B. Niu, Y. Tang, H. Luo, Z. Li, X. Yu, X. Wang, C. Jiang, Y. Qiao, S-G Sun. *Nanoscale*, **2023**, 15, 17751-17757.

[10] J. Liu, Y. Li, Y. Ding, L. Wu, J. Qin, T. Chen, C. Meng, F. Zhou, X. Ma, Z. Wu. *Angew. Chem. Int. Ed*. **2025**, 64, e202421107.

[11] F. Liu, M. Xue, T. Hu, T. Yao, C. Xu, L. Sheng, H. Dou, X. Zhang. *J. Phys. Chem. Lett.* **2024**, 15, 5738-5746.

[12] Q. Zhang, S. Rao, S. Vummaleti, E. Poh, W. Dai, X. Cui, J. Wu, J. Zhang, W. Chen. *Adv. Energy Mater.* **2022**, 12, 2200580.

[13] Q. Xiong, G. Huang, X. Zhang. *Angew. Chem. Int. Ed*. **2020**, 59, 19311-19319.

[14] H. Wan, Y. Sun, Z. Li, W. Wang, Y. Zhu, Y. Qian. *Energy Storage Mater.* **2021***,* 40, 159-165.
